# IRF3-mediated pathogenicity in a murine model of human hepatitis A

**DOI:** 10.1371/journal.ppat.1009960

**Published:** 2021-09-30

**Authors:** Lu Sun, You Li, Ichiro Misumi, Olga González-López, Lucinda Hensley, John M. Cullen, David R. McGivern, Mami Matsuda, Ryosuke Suzuki, Ganes C. Sen, Asuka Hirai-Yuki, Jason K. Whitmire, Stanley M. Lemon

**Affiliations:** 1 Lineberger Comprehensive Cancer Center, The University of North Carolina at Chapel Hill, Chapel Hill, North Carolina, United States of America; 2 Department of Genetics, The University of North Carolina at Chapel Hill, Chapel Hill, North Carolina, United States of America; 3 College of Veterinary Medicine, North Carolina State University, Raleigh, North Carolina, United States of America; 4 Department of Medicine, The University of North Carolina at Chapel Hill, Chapel Hill, North Carolina, United States of America; 5 Department of Virology II, National Institute of Infectious Diseases, Musashimurayama-shi, Tokyo, Japan; 6 Department of Inflammation and Immunity, Lerner Research Institute, Cleveland Clinic, Cleveland, Ohio, United States of America; 7 Management Department of Biosafety and Laboratory Animal, National Institute of Infectious Diseases, Musashimurayama-shi, Tokyo, Japan; 8 Department of Microbiology & Immunology, The University of North Carolina at Chapel Hill, Chapel Hill, North Carolina, United States of America; Duke University School of Medicine, UNITED STATES

## Abstract

HAV-infected *Ifnar1*^-/-^ mice recapitulate many of the cardinal features of hepatitis A in humans, including serum alanine aminotransferase (ALT) elevation, hepatocellular apoptosis, and liver inflammation. Previous studies implicate MAVS-IRF3 signaling in pathogenesis, but leave unresolved the role of IRF3-mediated transcription versus the non-transcriptional, pro-apoptotic activity of ubiquitylated IRF3. Here, we compare the intrahepatic transcriptomes of infected versus naïve *Mavs*^-/-^ and *Ifnar1*^-/-^ mice using high-throughput sequencing, and identify IRF3-mediated transcriptional responses associated with hepatocyte apoptosis and liver inflammation. Infection was transcriptionally silent in *Mavs*^-/-^ mice, in which HAV replicates robustly within the liver without inducing inflammation or hepatocellular apoptosis. By contrast, infection resulted in the upregulation of hundreds of genes in *Ifnar1*^-/-^ mice that develop acute hepatitis closely modeling human disease. Upregulated genes included pattern recognition receptors, interferons, chemokines, cytokines and other interferon-stimulated genes. Compared with *Ifnar1*^-/-^ mice, HAV-induced inflammation was markedly attenuated and there were few apoptotic hepatocytes in livers of infected *Irf3*^*S1/S1*^*Ifnar1*^*-/-*^ mice in which IRF3 is transcriptionally-inactive due to alanine substitutions at Ser-388 and Ser-390. Although transcriptome profiling revealed remarkably similar sets of genes induced in *Irf3*^*S1/S1*^*Ifnar1*^-/-^ and *Ifnar1*^-/-^ mice, a subset of genes was differentially expressed in relation to the severity of the liver injury. Prominent among these were both type 1 and type III interferons and interferon-responsive genes associated previously with apoptosis, including multiple members of the ISG12 and 2’-5’ oligoadenylate synthetase families. *Ifnl3* and *Ifnl2* transcript abundance correlated strongly with disease severity, but mice with dual type 1 and type III interferon receptor deficiency remained fully susceptible to liver injury. Collectively, our data show that IRF3-mediated transcription is required for HAV-induced liver injury in mice and identify key IRF3-responsive genes associated with pathogenicity, providing a clear distinction from the transcription-independent role of IRF3 in liver injury following binge exposure to alcohol.

## Author summary

Hepatitis A is a common and potentially serious disease involving inflammation and liver cell death resulting from infection with the picornavirus, hepatitis A virus (HAV). The pathogenesis of the disease is incompletely understood. Here, we have profiled changes in the RNA transcriptome of livers from mice with various genetic deficiencies in the innate immune response to HAV. We show that the liver injury associated with HAV infection in these mice results from the induction of genes under transcriptional control of interferon regulatory factor 3 (IRF3). We use high-throughput RNA sequencing to identify sets of genes induced in mice with wild-type versus transcriptionally-incompetent IRF3, rule out roles for type III interferons and IFIT proteins in disease pathogenesis, and identify genes with intrahepatic expression correlating closely with HAV-mediated liver pathology.

## Introduction

Although viral hepatitis is an important cause of human morbidity and mortality worldwide, the absence of tractable small animal models for hepatotropic human viruses has handicapped efforts to understand anti-viral immunity and inflammatory responses within the liver [1,2]. Recent studies have shown, however, that *Ifnar1*^*-/-*^ mice lacking expression of the type I interferon (IFN) receptor are highly permissive for infection with hepatitis A virus (HAV), the causative agent of type A hepatitis in humans [3–5]. These mice experience an hepatotropic HAV infection associated with elevated levels of liver-specific enzymes in the serum, fecal shedding of virus through the biliary track, and mixed inflammatory cell infiltrates surrounding apoptotic infected hepatocytes in the liver. The clinical course of HAV infection in these mice and histopathologic evidence of HAV-induced hepatocellular apoptosis closely mirror HAV infections in humans and experimentally-infected chimpanzees [6,7]. These mice thus offer unique opportunities for investigating pathogenic mechanisms underlying liver injury in acute viral hepatitis.

The inflammatory liver injury observed in HAV-infected *Ifnar1*^*-/-*^ mice appears to result from cell-intrinsic, innate immune responses to the virus induced via RIG-I-like pattern recognition receptors (RLRs) [3]. *Mavs*^*-/-*^ and *Irf3*^*-/-*^*Irf7*^*-/-*^ mice, both of which lack the signaling downstream of RLRs required for virus-induced IFN synthesis, are just as permissive for infection as *Ifnar*1^-/-^ mice, but they do not develop liver disease when infected with HAV. Neither *Mavs*^*-/-*^ mice nor *Irf3*^*-/-*^*Irf7*^*-/-*^ mice develop elevated serum alanine aminotransferase (ALT) activities or hepatic inflammation when infected, despite having equal or greater quantities of virus within the liver [3]. Numerous apoptotic hepatocytes are present in infected *Ifnar1*^*-/-*^ liver, but few if any are found in infected *Mavs*^*-/-*^ tissue [3]. Consistent with the notion that hepatitis results from cell-intrinsic responses, prior depletion of CD4+ or CD8+ T cells, NK/NK-T cells, or phagocytic macrophages has no impact on the development of hepatitis following intravenous challenge of *Ifnar1*^*-/-*^ mice with HAV [3].

Cell-intrinsic responses to HAV mediated by mitochondrial antiviral signaling protein (MAVS) and interferon regulatory factor (IRF3) result in the expression of multiple ‘interferon-stimulated genes’ (ISGs) in infected *Ifnar1*^*-/-*^ mice, despite the absence of paracrine IFN-signaling through the type I IFN receptor [3]. These genes are likely induced directly by transcriptionally-active, phosphorylated IRF3 [8]. Phosphorylated IRF3 can be detected in livers of infected *Ifnar1*^*-/-*^ mice [3], as can transcripts of IRF3-responsive ISGs encoding potentially pro-apoptotic proteins such as phorbol-12-myristate-13-acetate-induced protein 1 (PMAIP, a.k.a Noxa) [3]. However, activated IRF3 can also induce apoptosis directly through a transcription-independent pathway involving its direct interaction with mitochondrial Bax [9]. Thus, either mechanism of IRF3-mediated apoptosis could be important for HAV induced liver disease.

There is increasing evidence that IRF3 plays a pivotal role in liver injury resulting from a variety of causes, including chemical and viral exposures and non-alcoholic fatty liver disease (NAFLD) [10–12]. Hepatocytes are highly sensitive to Fas ligation, which induces apoptosis and the expression of chemokines and other inflammatory mediators [13]. Deleting *Irf3* protects against Fas-mediated liver injury as well as hepatocellular apoptosis resulting from acute ethanol or CCl_4_ exposure in mice [10,14]. IRF3 mediates pathology independently of downstream IFN signaling, as *Ifnar1*^*-/-*^ mice are not protected against ethanol-related liver injury [10,15] or HAV-induced liver disease [3]. Acute alcohol exposure induces IRF3 to interact with mitochondrial Bax [15], which suggests that ethanol-induced hepatocyte apoptosis might result from the transcription-independent activity of ubiquitylated IRF3 [9]. Recent studies are consistent with this hypothesis, as *Irf3*^*S1/S1*^ mice, with Ser-to-Ala substitutions rendering IRF3 transcriptionally incompetent, remain susceptible to liver injury following acute-on-chronic exposure to ethanol, whereas *Irf3*^*-/-*^ mice completely deficient in IRF3 are protected from liver injury [16].

Here, we report a series of experiments aimed at further elucidating the mechanism of liver injury in HAV-infected *Ifnar1*^-/-^mice. We profile changes in the intrahepatic transcriptomes of HAV-infected *Mavs*^-/-^ and *Ifnar1*^-/-^ mice, and investigate the role played by transcriptional activation of IRF3 in the pathogenesis of acute hepatitis A. We demonstrate that HAV-mediated hepatocellular apoptosis requires transcriptional activation of IRF3 by phosphorylation at Ser^388/399^, distinguishing IRF3-dependent HAV liver injury from IRF3-mediated alcohol-related disease, and identify IRF3-responsive genes for which the level of intrahepatic expression correlates closely with the severity of the liver injury.

## Results

### Transcriptional profiling of HAV-infected *Mavs*^-/-^ and *Ifnar1*^-/-^ mice

**Fig 1:**
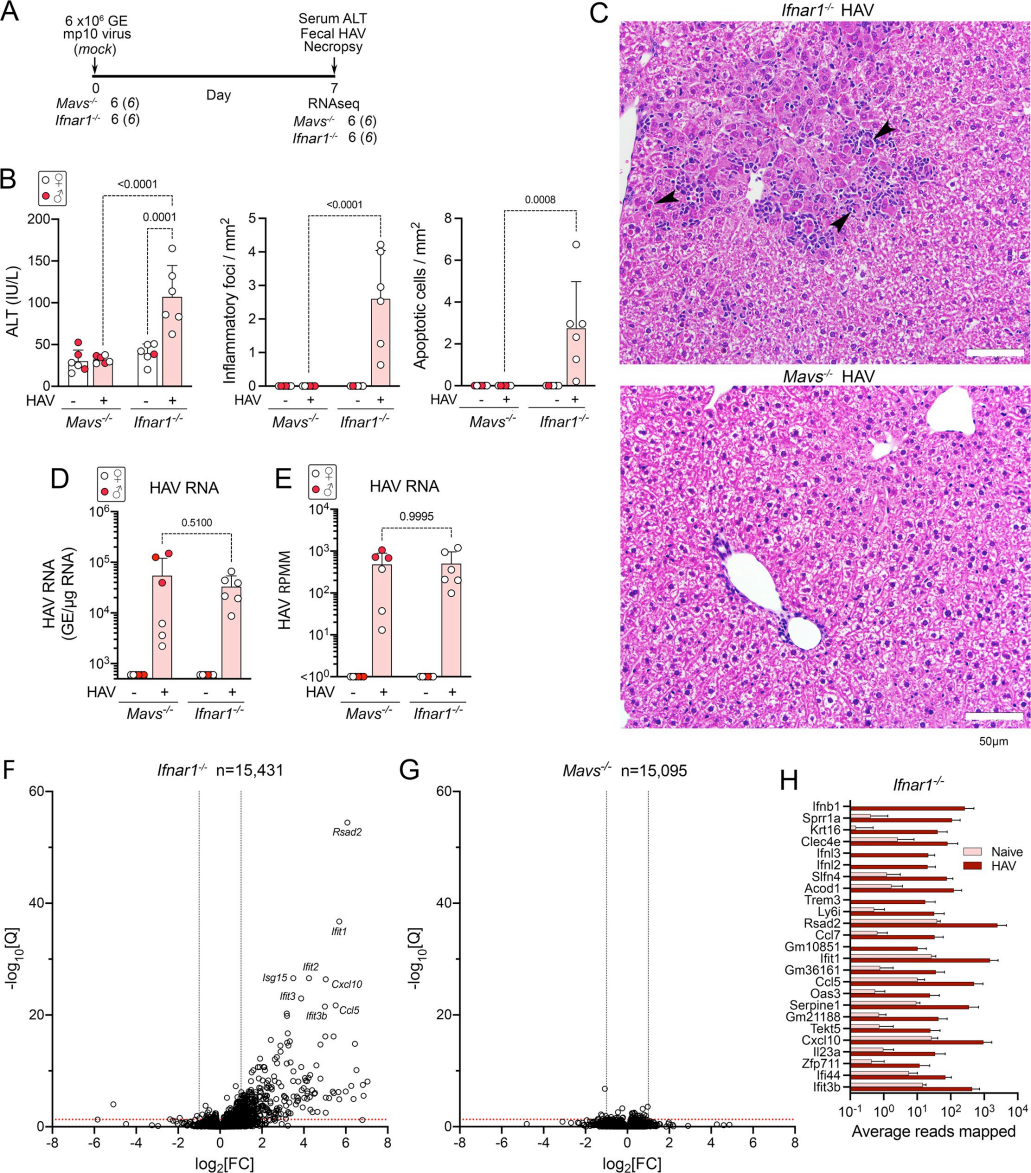
Infection-induced changes in the intrahepatic transcriptome of HAV-infected *Mavs*^*-/-*^ and *Ifnar1*^*-/-*^ mice. **(A)** Experimental scheme showing numbers of mice inoculated with 6 x10^6^ genome equivalents (GE) of HM175-mp10 virus. **(B)** (*left*) Serum alanine transaminase (ALT) activities in sera of HAV-infected and mock-infected (naïve) mice 7 days after inoculation (dpi); (*center*) inflammatory foci; and, (*right*) apoptotic cells identified in H&E-stained liver sections from infected (+) and HAV-naive (-) mice. Liver sections were reviewed by a veterinary hepatic pathologist who was blinded to the inoculum for each mouse. Histopathology results are shown as the mean number of inflammatory foci or apoptotic cells per mm^2^ area derived from scoring of 20 random 40x microscopic fields of view. In this and all other panels, male animals are indicated by solid red symbols, and female animals by open symbols. **(C)** Representative H&E stained liver sections. Arrowheads indicate apoptotic hepatocytes surrounded by inflammatory immune cells. **(D)** HAV RNA abundance in liver tissue determined by RT-qPCR. **(E)** HAV RNA transcript abundance determined by high-throughput RNA sequencing. RPMM = reads per million reads mapped. **(F)** Volcano plot showing fold-change (FC) and significance of changes in intrahepatic transcripts of genes with at least 10 reads mapped in RNA from naïve versus HAV-infected *Ifnar*^*-/-*^ mice following conditioning for sex and RNAseq batch. Q = adjusted p-value. Red horizontal line indicates Q = 0.05. **(G)** Similar plot of fold-change in transcript abundance in infected *Mavs*^*-/-*^ mice. See also **S1D Fig**. **(H)** Mean reads mapped in liver tissue from naïve and HAV-infected female *Ifnar*^*-/-*^ mice for the 25 differentially expressed transcripts with the greatest fold-change in abundance. Genes are rank-ordered according to the fold-change between naïve and infected. Error bars in all panels indicate s.d.; p-values are shown for 2-way ANOVA with Šidák’s test for multiple comparisons.

Cohorts of *Mavs*^-/-^ and *Ifnar1*^-/-^ mice (6 each) were inoculated intravenously with liver homogenate from an infected *Mavs*^-/-^ mouse containing 6 x10^6^ genome equivalents (GE) of 10^th^ mouse-passage virus (HM175-mp10 inoculum), or mock-infected by inoculation of an equal volume of PBS **(Fig 1A)**. HAV-infected and mock-infected animals were drawn from the same litters. Mice were necropsied 7 days post-inoculation (dpi). Serum ALT levels were modestly elevated at necropsy in infected *Ifnar1*^-/-^ mice (mean 107 IU/L ± 38 s.d., compared with a mean 39 IU/L ± 11 s.d. in uninfected *Ifnar1*^-/-^ mice, p = 0.0001) **(Fig 1B)**. Apoptotic hepatocytes and scattered inflammatory cell infiltrates were present in H&E-stained liver sections from these animals **(Fig 1B and 1C)**. By contrast, neither ALT elevation nor histopathologic changes were observed in the infected *Mavs*^-/-^ mice (**Fig 1B and 1C**), despite similar intrahepatic viral load **(Fig 1D)**. These findings recapitulate earlier observations of HAV infections in these genetically-modified mice [3].

To characterize changes in the intrahepatic transcriptome associated with HAV infection, RNA extracted from the livers of these mice was subjected to high-throughput sequencing. A mean of 5.09 x10^7^ individual reads (representing 61–80% of all reads) in each sample mapped uniquely to the mouse genome **(S1A Table)**. Transcripts were identified from a total of 15,095 host genes in *Mavs*^-/-^ mice and 15,431 genes in *Ifnar1*^-/-^ mice. Comparable numbers of reads mapped to the HAV genome in samples from infected *Mavs*^-/-^ and *Ifnar1*^-/-^ mice (**Fig 1E**). Principal component analysis (PCA) identified sex and sequencing batch as the major contributors to variance in transcript abundance (samples were sequenced in two separate batches) **(S1A Fig)**. Infection status contributed only ~9% to the overall variance, with PCA failing to distinguish infected from uninfected *Mavs*^-/-^ mice (all of the infected *Ifnar1*^*-/-*^ mice were females). While surprising, previous studies have shown that there is extensive sexual dimorphism in the intrahepatic transcriptome, driven in part by sex differences in growth hormone-regulated STAT5 and the hepatic estrogen receptor [17,18]. Nonetheless, after conditioning for sex and sequencing batch, a total of 376 genes were significantly differentially expressed (>2-fold change with *p*-value <0.05 after adjustment by the Benjamini-Hochberg method) in infected versus uninfected *Ifnar1*^-/-^ mice (**Table 1**). Of these genes, 372 were upregulated and only 4 downregulated (**Figs 1F, S1B, and S1C**). By contrast, in infected *Mavs*^-/-^ mice, there were no genes comparably upregulated, and 6 genes downregulated (**Figs 1G** and **S1D and Table 1**).

**Table 1:** High-throughput sequencing of intrahepatic RNA from HAV-infected mice.

Sequencing batch run	Mouse genotype	n*	Virus Inoculum	Days p.i.	Mean HAV RNA^†^	ALT^‡^	Differentially-expressed transcripts^¶^			
							2x ↑	2x ↓	4x ↑	4x ↓
1,2	*Mavs* ^*-/-*^	6	mp10	7	5.4 x10^4^	32	0	6	0	0
1,2	*Ifnar1* ^*-/-*^	6	mp10	7	3.3 x10^4^	107	372	4	145	2
3	*Ifnar1* ^*-/-*^	4	mp6	14	3.5 x10^5^	195	1028	74	380	10
3	*Irf3* ^*S1/S1*^ *Ifnar1* ^*-/-*^	4	mp6	14	4.6 x10^5^	26	182	24	66	3

*Number of mice in each naïve and infected cohort

^†^Intrahepatic viral RNA (genome equivalents/μg total RNA)

^**‡**^Serum ALT (IU/L) on day 7 p.i.

^**¶**^HAV-infected versus naïve animals with Q <0.

Genes encoding interferons (*Ifnb*, *Ifnl2* and *Ifnl3*), chemokines (*Ccl7*, *Ccl5*, and *Cxcl10*), and ISGs (*Ifit1*, *Ifit2*, *Ifit3b*, *Oas3*, *Ifi44*, *Rsad2*, and *Isg15*, among others) were prominently represented among those induced by infection in *Ifnar1*^*-/-*^ mice (**Fig 1F and 1H and S1B Table**). None of these were upregulated in *Mavs*^*-/-*^ mice (**S1E Fig and S1C Table**). Consistent with earlier studies [3], fluorescent *in situ* hybridization of liver tissue indicated that *Ccl5* transcripts were expressed primarily within HAV-infected hepatocytes, which were easily distinguished from infiltrating lymphocytes and macrophages by their generous cytoplasm (**S2 Fig**). HAV RNA was readily detected in infected liver from both *Ifnar1*^*-/-*^ and *Mavs*^*-/-*^ mice, but present in a patchy distribution involving less than 10–25% of hepatocytes (**S2 Fig)**. Geneset Enrichment Analysis (GSEA) was consistent with a profound activation of the innate immune system, including a highly significant match with the hallmark complement component gene set (**S1F Fig**). As suggested previously, this striking transcriptional response to HAV infection in *Ifnar1*^*-/-*^ mice likely arises from MAVS-dependent transcriptional activation of IRF3 [3].

### HAV infection in *Ifnar1*^*-/-*^ mice with locus-wide knockout of *Ifit* genes

**Fig 2:**
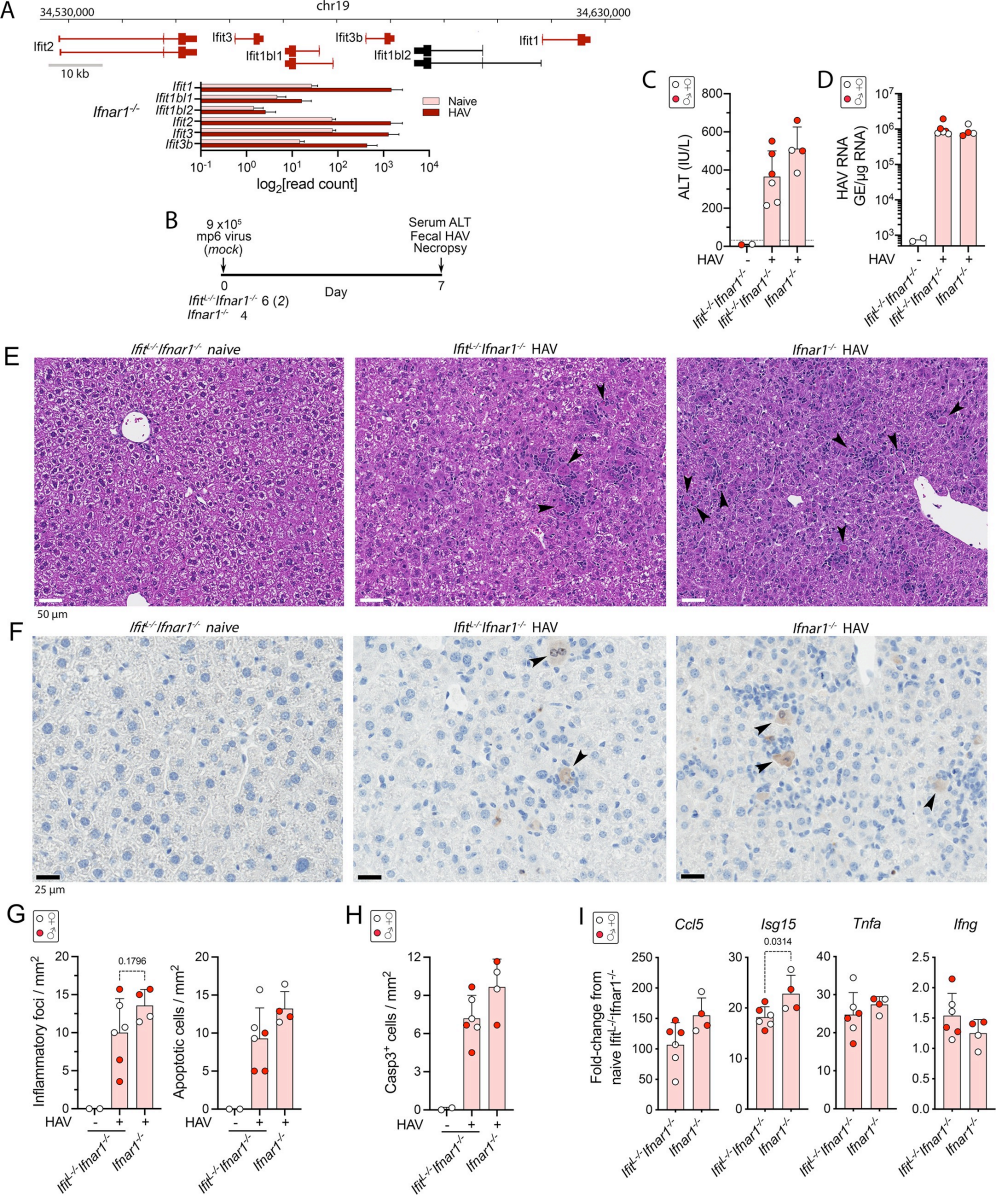
HAV infection of *Ifit* locus knockout *Ifit*^*L-/-*^*Ifnar1*^*-/-*^ and *Ifnar1*^*-/-*^ mice. **(A)** *Ifit* gene locus in chromosome 19, showing exons and introns related to major transcripts encoding IFIT proteins. Transcripts identified by high-throughput sequencing in naïve and HAV-infected mouse liver are colored red. **A**t the bottom are shown the normalized *Ifit* locus transcript read counts in livers of naïve and HAV-infected female *Ifnar1*^*-/-*^ mice (n = 4). **(B)** Experimental scheme showing numbers of HAV- and mock-infected *Ifit*^*L-/-*^*Ifnar1*^*-/-*^ and *Ifnar1*^*-/-*^ mice. Mice were necropsied 7 days after i.v. inoculation (dpi) of 9 x10^5^ genome equivalents (GE) of HM175-mp6 virus. **(C)** Serum ALT activities in mock-infected and HAV-infected *Ifit*^*L-/-*^*Ifnar1*^*-/-*^ mice versus HAV-infected *Ifnar1*^*-/-*^ mice. In this and other panels, male animals are indicated by solid red symbols, and female animals by open symbols. **(D)** Intrahepatic HAV RNA abundance 7 dpi determined by RT-qPCR GE = genome equivalents. **(E)** Representative H&E stained liver sections from naïve and HAV-infected *Ifit*^*L-/-*^*Ifnar1*^*-/-*^ and *Ifnar1*^*-/-*^ mice. **(F)** Representative immunohistochemically stained liver sections demonstrating cells with intracellular cleaved caspase 3 (arrows). **(G)** Foci of inflammation (*left*) and apoptotic hepatocytes (*right*) observed by a blinded liver pathologist in H&E-stained sections of liver collected at necropsy of naïve and infected mice. **(H)** Mean number of hepatocytes staining positively for cleaved caspase 3 per mm^2^ in liver tissue collected at necropsy. **(I)** Cytokine transcript abundance in liver tissue collected at necropsy. Error bars in all panels indicate s.d.; p-values are shown only when <0.05 in 2-way comparisons by two-sided t test or ANOVA with Šidák’s test for multiple comparisons.

Multiple members of the IFIT (interferon-induced protein with tetratricopeptide repests) family of ISGs (*Ifit1*, *Ifit2*, *Ifit3*, and *Ifit3b* were among those most highly induced in the infected *Ifnar1*^*-/-*^ mice (**Fig 1F)**. This family is comprised of 6 proteins with tetratricopeptide repeats encoded by genes clustered in chromosome 19, 4 of which were induced over 10-fold **(Fig 2A and 2B)**. These ISGs are diverse in sequence and exert a variety of antiviral activities by interacting with both viral RNAs and proteins [19]. The antiviral activity of IFITs against HAV has not been studied previously, and little is known about their activity within the murine liver. Although distinct in sequence from its murine ortholog, human IFIT2 promotes Bcl family member (Bax and Bak) dependent mitochondrial apoptosis when expressed in murine cells [20]. To determine whether the induction of IFITs contribute to hepatocellular apoptosis and inflammation in infected *Ifnar1*^-/-^ mice [3], we challenged mice crossed with a genetic knockout of the entire chromosome 19 IFIT locus (*Ifit*^*L*-/-^ mice) [21].

Cohorts of 4–6 *Ifit*^*L*-/-^*Ifnar1*^-/-^ or *Ifnar1*^-/-^ mice were inoculated intravenously with 9 x10^5^ GE of 6^th^ mouse-passage virus (HM175-mp6) and necropsied 7 days later. This inoculum is notably more virulent than the HM175-mp10 inoculum used to infect mice in the experiment shown in Fig 1, and caused substantially higher serum ALT elevations in *Ifnar1*^-/-^ mice on day 7 post infection (p.i.) (**Fig 2C**). Although ALT levels tended to be higher in infected *Ifnar1*^-/-^ versus *Ifit*^*L*-/-^*Ifnar1*^-/-^ mice, this difference did not achieve statistical significance. The intrahepatic viral load was similar at necropsy on day 14 (**Fig 2D**), and liver sections from both *Ifit*^*L*-/-^*Ifnar1*^-/-^ and *Ifnar1*^-/-^ mice demonstrated moderate inflammatory cell infiltrates with apoptotic hepatocytes scattered throughout the parenchyma **(Fig 2E)**. Immunohistochemical staining for cleaved caspase 3 revealed numerous apoptotic hepatocytes in both types of mice **(Fig 2F)**. While the histopathologic changes tended to be milder in the *Ifit*^*L*-/-^*Ifnar1*^-/-^ animals, there was clear overlap with *Ifnar1*^-/-^ mice and this difference did not achieve statistical significance (**Fig 2G and 2H**). Similar trends existed in *Ccl5* and *Isg15* (p = 0.0314) transcript levels (**Fig 2I)**, suggesting less IRF3 activation in *Ifit*^*L*-/-^*Ifnar1*^-/-^ mice despite similar intrahepatic viral loads (**Fig 2D**). *Tnfa* and *Ifng* responses, both of which tend to peak later in the infection [3], were similar in *Ifit*^*L*-/-^*Ifnar1*^-/-^ and *Ifnar1*^-/-^ mice. We conclude that IFITs make no more than a minor contribution to the liver injury, including hepatocellular apoptosis, associated with HAV infection in *Ifnar1*^-/-^ mice, and although robustly induced have no anti-HAV activity in the murine liver.

### HAV pathogenesis in mice with transcriptionally-incompetent IRF3

**Fig 3:**
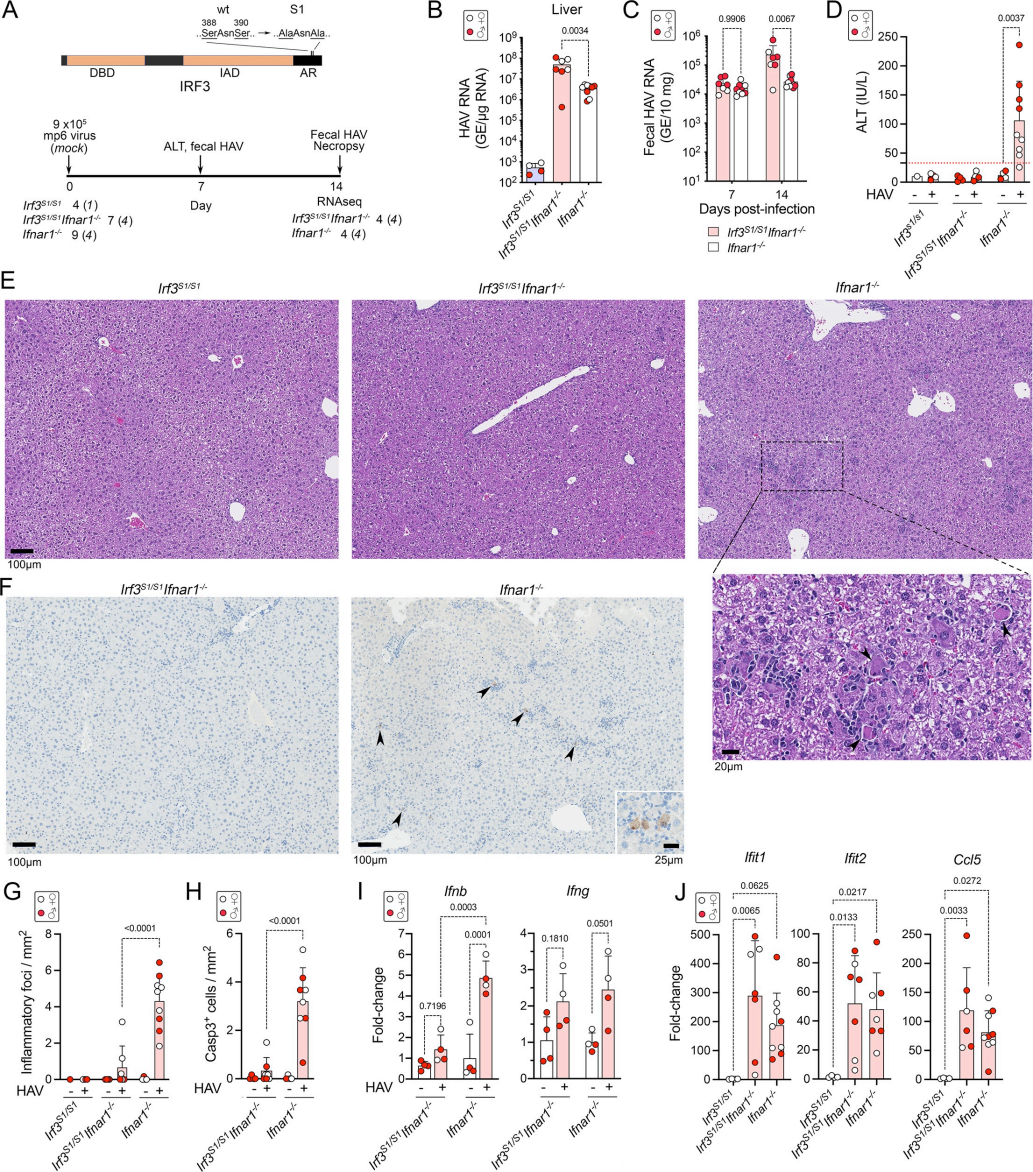
Infectious HAV challenge of *Irf3*^*S1/S1*^, *Irf3*^*S1/S1*^*Ifnar1*^*-/-*^, and *Ifnar1*^*-/-*^ mice. **(A)** (*top*) IRF3 protein structure showing location of Ser-to-Ala substitutions in *Irf3*^*S1/S1*^ mice that render the protein transcriptionally inactive. DBD, DNA-binding domain; IAD, IRF-association domain; AR, autoinhibitory region. (*bottom*) Experimental scheme. A total of 4 *Irf3*^*SI/SI*^ mice, 7 *Irf3*^*S1/S1*^*Ifnar1*^*-/-*^ mice, and 9 *Ifnar1*^*-/-*^ mice were infected with 9 x10^5^ HM175-mp6 virus in two independent experiments. Liver tissue collected at necropsy 14 days later from representative infected and mock-infected *Irf3*^*S1/S1*^*Ifnar1*^*-/-*^ and *Ifnar1*^*-/-*^ mice (4 each) were selected for high-throughput RNA sequencing. *Irf3*^*SI/SI*^ mice showed no evidence of infection and these livers were not subjected to sequencing. **(B)** HAV RNA in livers of mice determined by RT-qPCR 14 days post-inoculation (dpi). In all panels, male animals are indicated by solid red symbols, and female animals by open symbols. **(C)** Fecal HAV RNA 7 and 14 dpi. **(D)** Serum ALT in *Irf3*^*S1/S1*^, *Irf3*^*S1/S1*^*Ifnar1*^*-/-*^, and *Ifnar1*^*-/-*^ mice 7 dpi. **(E)** Representative H&E-stained sections of (*left* to *right*) *Irf3*^*S1/S1*^, *Irf3*^*S1/S1*^*Ifnar1*^*-/-*^, and *Ifnar1*^*-/-*^ mouse liver 14 dpi. Arrowheads in the enlarged image of *Ifnar1*^*-/-*^ liver indicate apoptotic hepatocytes. **(F)** Representative immunohistochemically stained liver sections showing detection of cleaved caspase 3 in liver from (*left*) *Irf3*^*S1/S1*^*Ifnar1*^*-/-*^ and (*right*) *Ifnar1*^*-/-*^ mice 14 dpi. **(G)** Number of inflammatory foci per mm^2^ in H&E-stained liver sections. **(H)** Mean number of hepatocytes staining positively for cleaved caspase 3 (Casp3) per mm^2^ in liver sections by immunohistochemistry. **(I)** RT-qPCR determination of the fold-change in intrahepatic *Ifnb* and *Ifng* transcript abundance in groups of infected and mock-infected *Irf3*^*S1/S1*^*Ifnar1*^*-/-*^ and *Ifnar1*^*-/-*^ mice selected for high-throughput sequencing (n = 4 in each group). (**J**) RT-qPCR determination of infection-induced changes in intrahepatic *Ifit1*, *Ifit2* and *Ccl5* transcript levels in mice from both experiments. Error bars in all panels indicate s.d.; p-values are shown for 2-way comparisons by two-way ANOVA with Šidák’s test for multiple comparisons.

The MAVS-dependent phosphorylation of IRF3 in HAV-infected *Ifnar1*^-/-^ mice induces pro-apoptotic and pro-inflammatory gene expression [3] (**Fig 1F and S1B Table**). However, RIG-I-like receptor (RLR)-induced signaling through MAVS and TRAF2/3/6 can also lead to the linear ubiquitylation of IRF3, which then interacts with mitochondrial Bax to induce apoptosis [9,22]. This alternative IRF3-mediated mechanism of apoptosis, known as the “RLR-induced IRF3-mediated pathway of apoptosis” (RIPA), is not dependent upon the phosphorylation of IRF3 at serine residues near its C-terminus (Ser-388 and Ser-390) that activate it transcriptionally. RIPA has been shown to provide protection against Sendai virus infection in genetically modified *Irf3*^*S1/S1*^ mice, in which IRF3 is transcriptionally incompetent due to alanine substitutions at Ser-388 and Ser-390 [9], and has also been shown to promote liver injury in alcohol-exposed mice [16] **(Fig 3A)**. We challenged *Irf3*^*S1/S1*^ mice with HAV to ascertain whether hepatocellular apoptosis and liver injury associated with HAV infection requires IRF3-directed transcription, or alternatively might result from RIPA mediated by the direct interaction of ubiquitylated IRF3 with Bax.

Cohorts of 4–9 *Irf3*^*S1/S1*^, *Irf3*^*S1/S1*^*Ifnar1*^-/-^ or *Ifnar1*^-/-^ mice were inoculated i.v. with 9 x10^5^ GE HM175-mp6 virus or sham control. HAV failed to establish infection in *Irf3*^*S1/S1*^ mice, indicating that eliminating the transcriptional activity of IRF3 does not by itself render mice permissive for the virus (**Fig 3B**). These results mirror previous studies in which *Irf3*^*-/-*^ mice lacking IRF3 expression were not susceptible to infection [3]. By contrast, *Irf3*^*S1/S1*^*Ifnar1*^-/-^ mice were permissive for infection and shed more virus in feces than *Ifnar1*^-/-^ mice on days 7 and 14 p.i. (**Fig 3C**). Viral load was also higher in the liver of *Irf3*^*S1/S1*^*Ifnar1*^-/-^ mice on day 14 (**Fig 3B)**. Thus, although loss of IRF3 transcription does not render mice permissive for infection, it does result in reduced virus control in mice lacking the type I IFN receptor.

In sharp contrast to the greater intrahepatic viral load in *Irf3*^*S1/S1*^*Ifnar1*^-/-^ mice, there was no elevation of serum ALT levels in these mice (**Fig 3D**). Consistent with this, only rare inflammatory cell infiltrates and cleaved caspase 3-positive apoptotic hepatocytes were observed in livers from infected *Irf3*^*S1/S1*^*Ifnar1*^-/-^ mice, whereas both were abundant in infected *Ifnar1*^-/-^ mice (**Fig 3E–3H**). IFNβ (*Ifnb*) transcripts were minimally induced in the liver of *Irf3*^*S1/S1*^*Ifnar1*^-/-^ mice compared with *Ifnar1*^-/-^ mice, whereas there was no difference in IFNγ (*Ifng*) transcripts which were minimally elevated (about 2-fold) in both (**Fig 3I**). Thus, hepatocyte apoptosis, hepatic inflammation and HAV-induced IFNβ expression are highly dependent upon the phosphorylation of IRF3 in HAV-infected mice. Despite these differences, and the marked difference in liver injury, transcripts for chemokines and ISGs such as *Ccl5*, *Ifit1*, and *Ifit2* were equivalently induced in *Irf3*^*S1/S1*^*Ifnar1*^-/-^ and *Ifnar1*^-/-^ mice (**Fig 3J**). These genes were not transcriptionally induced in *Irf3*^*S1/S1*^ mice.

### Transcriptional profiling of HAV-infected *Irf3*^*S1/S1*^*Ifnar1*^-/-^ mice

**Fig 4:**
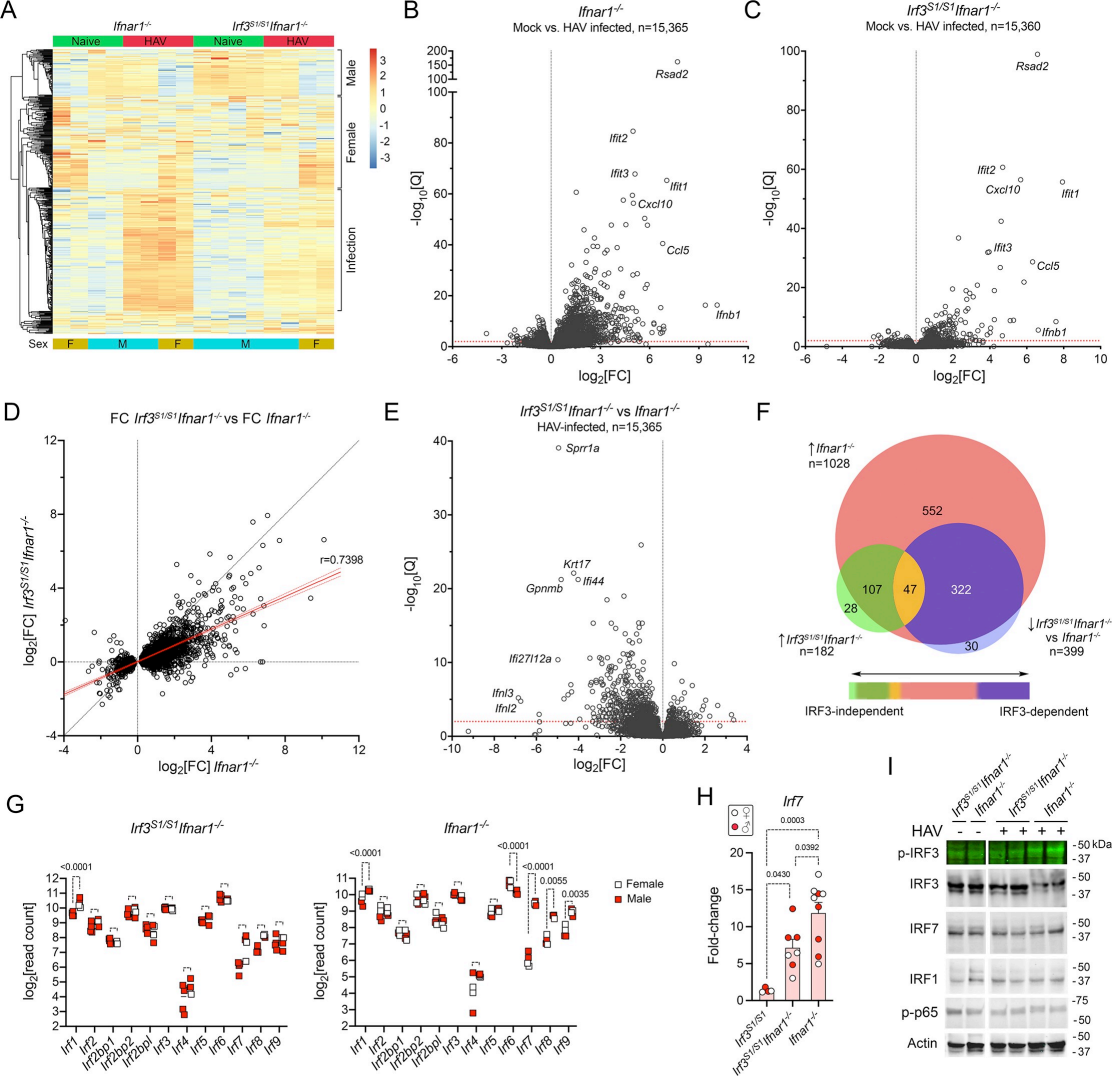
Changes in the intrahepatic transcriptome of HAV-infected *Irf3*^*S1/S1*^*Ifnar1*^*-/-*^ mice. **(A)** Heat map showing changes in abundance of the top 500 differentially-expressed genes (ranked by variance of standardized read counts) in naïve and HAV-infected *Irf3*^*S1/S1*^*Ifnar1*^*-/-*^ and *Ifnar1*^*-/-*^ mice; n = 4 in each group selected for high-throughput RNA sequencing. Transcripts differentially expressed in relation to sex or HAV infection are noted on the right. The scale on the right represents z-score. **(B, C)** Volcano plots showing fold-change (fc) and significance of changes in transcripts of genes with at least 10 reads mapped in RNA from naïve versus HAV-infected liver tissue from (B) *Ifnar1*^*-/-*^ and (C) *Irf3*^*S1/S1*^*Ifnar1*^*-/-*^ mice. Q = adjusted p-value. **(D)** Correlation of fold-change (log_2_[FC]) observed in abundance of 2,503 differentially expressed transcripts in HAV-infected *Irf3*^*S1/S1*^*Ifnar1*^*-/-*^ mice versus *Ifnar1*^*-/-*^ mice. Red line = simple linear regression, ±95% C.I.; slope = 0.4415; Spearman *r* = 0.7398, p<0.0001. **(E)** Volcano plot showing fold-change and significance of infection-induced differences in intrahepatic RNA transcripts in *Irf3*^*S1/S1*^*Ifnar1*^*-/-*^ versus *Ifnar1*^*-/-*^ mice. **(F)** Venn diagram showing overlap of genes upregulated by infection in *Ifnar1*^*-/-*^ and *Irf3*^*S1/S1*^*Ifnar1*^*-/-*^ mice. **(G)** Normalized read counts of interferon regulatory factor transcripts in mock versus HAV-infected (*left*) *Irf3*^*S1/S1*^*Ifnar1*^*-/-*^ and (*right*) *Ifnar1*^*-/-*^ mice. Significance determined by two-way ANOVA with Šídák’s multiple comparisons test. **(H)** Infection induced fold-change in Irf7 transcript abundance determined by RT-qPCR. Fold increase for *Irf3*^*S1/S1*^ was 1.38 ± 0.31 s.d. Solid red symbols represent male animals. **(I)** Immunoblots of interferon regulatory factors, including phosphorylated IRF3 (p-IRF3, Ser396), and phosphorylated RELA (p-p65) in extracts of liver tissue from naïve and HAV-infected (*left*) *Irf3*^*S1/S1*^*Ifnar1*^*-/-*^ and (*right*) *Ifnar1*^*-/-*^ mice.

To better understand differences in the transcriptional response to HAV infection in *Irf3*^*S1/S1*^*Ifnar1*^-/-^ and *Ifnar1*^-/-^ mice, we sequenced RNA isolated from the livers of infected and naive animals of each genotype (n = 4 each) (**Fig 4A and Table 1**). Alignment statistics for the derived RNA reads (**S1D Table**) were similar to those described above, with a greater percentage of reads mapping to the HAV genome in livers from infected *Irf3*^*S1/S1*^*Ifnar1*^-/-^ than *Ifnar1*^-/-^ mice (**S3A Fig**). PCA suggested sex contributed ~57% of the variance in gene expression, with infection status contributing ~22% (**S3B Fig**). After conditioning for sex, a total of 1028 genes were significantly upregulated, and 74 down-regulated, by infection in *Ifnar1*^*-/-*^ mice (**Tables 1** and **S1E**). By contrast, only 182 genes were upregulated and 24 down-regulated in *Irf3*^*S1/S1*^*Ifnar1*^-/-^ mice (**Tables 1** and **S1F**). The greater number of genes that were significantly induced in the *Ifnar1*^-/-^ mice in this second transcriptome study compared to that shown in Fig 1 reflects the use of a more potent viral inoculum (HM175-mp6 vs. HM-175-mp10), a greater length of time since infection (14 versus 7 days), and consequently more severe liver injury (mean serum ALT 195 IU/L versus 107 IU/L). These differences notwithstanding, changes in the expression levels of individual upregulated genes correlated well in the two experiments **(S3C Fig)**. There was little difference in the transcriptomes of uninfected *Irf3*^*S1/S1*^*Ifnar1*^-/-^ and *Ifnar1*^-/-^ mice (**S3D Fig**).

The genes induced by HAV infection were remarkably similar in *Irf3*^*S1/S1*^*Ifnar1*^-/-^ and *Ifnar1*^-/-^ mice, with *Rsad2*, *Ifit1*, *Ifit2*, *Ifit3* and *Cxcl10* among those most prominently upregulated (**Fig 4B and 4C**). Strikingly, *Rsad2* (viperin) was the most highly induced gene in both studies of *Ifnar1*^-/-^ mice (**Figs 1F and 4B**), and also in *Irf3*^*S1/S1*^*Ifnar1*^-/-^ mice (**Fig 4C**). A strong positive correlation existed between the fold-change of individual transcripts in *Irf3*^*S1/S1*^*Ifnar1*^-/-^ and *Ifnar1*^-/-^ mice (Spearman *r* = 0.7398, p<0.0001) (**Fig 4D**). Nonetheless, a comparison of infected mice identified 399 genes that were >2-fold less expressed in *Irf3*^*S1/S1*^*Ifnar1*^-/-^ compared with *Ifnar1*^-/-^ mice (**Fig 4E and S1G Table**). These included 47 genes that were significantly induced by infection in both *Irf3*^*S1/S1*^*Ifnar1*^-/-^ and *Ifnar1*^-/-^ mice, and 322 induced only in *Ifnar1*^-/-^ animals (**Figs 4F** and **S3E**). Particularly notable among genes that were significantly less induced in the *Irf3*^*S1/S1*^*Ifnar1*^-/-^ mice were those encoding IFNλ2 (*Ifnl2*) and IFNλ3 (*Ifnl3*) **(Fig 4E**). These results thus establish sets of genes that are transcriptionally induced by HAV infection largely independent of IRF3 phosphorylation at Ser-388 or Ser-399, and others that are highly dependent upon phosphorylation of these IRF3 residues.

To gain insight into transcription factor(s) driving gene expression in infected *Irf3*^*S1/S1*^*Ifnar1*^-/-^ and *Ifnar1*^-/-^ mice, we sought evidence for transcriptional induction of members of the IRF family. Baseline expression prior to infection was similar in the two types of mice (**S3F Fig**). *Irf1*, *Irf7*, *Irf8*, and *Irf9* transcripts were significantly induced by infection in *Ifnar1*^-/-^ mice, whereas *Irf6* transcripts (abundant in uninfected mice) were significantly down-regulated (**Fig 4G**). The mechanism underlying the decrease in *Irf6* transcripts is uncertain, but IRF6 is known to function primarily in maintaining epidermal barrier function, not host responses to viral infection [23]. *Irf1* was significantly upregulated in infected *Irf3*^*S1/S1*^*Ifnar1*^-/-^ mice (**Fig 4G**). While *Irf7* transcript abundance was much lower than *Irf1*, *Irf7* was also upregulated when measured by RT-qPCR in a larger number of animals **(Fig 4H)**. suggesting IRF1 may drive most ISG expression in these animals. Immunoblots of liver tissues confirmed phosphorylation of IRF3 at Ser396 in infected *Ifnar1*^-/-^ mice, and the absence of this in infected *Irf3*^*S1/S1*^*Ifnar1*^-/-^ animals (**Fig 4I**). Efforts to document the presence of ubiquitylated IRF3 were unsuccessful. We observed no changes in the abundance or molecular mass of IRF7 or IRF1 in either type of mouse. Similarly, efforts to detect phosphorylated IRF7 were unsuccessful. Although IRF1 is regulated transcriptionally by NF-κB [24], there was no detectable increase in phospho-p65 (**Fig 4I**). Efforts to document these changes in transcription factors by immunoblotting were likely limited by technical difficulties quantifying these proteins in liver extracts, as well as the low proportion of hepatocytes infected with virus (<25%) **(S2B Fig**).

### Gene expression and liver histopathology in HAV-infected mice

**Fig 5:**
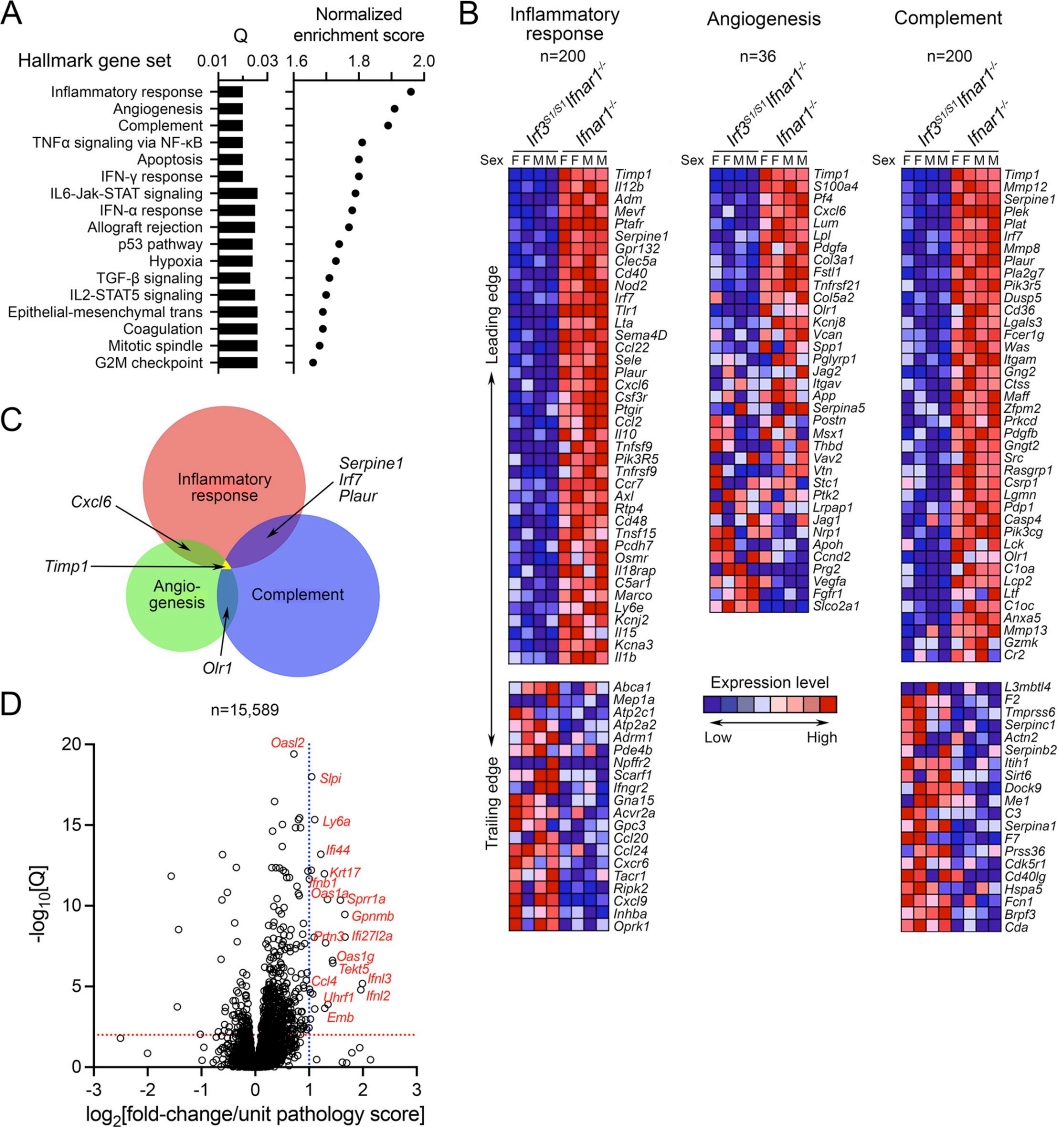
Differentially expressed intrahepatic transcripts in HAV-infected *Irf3*^*S1/S1*^*Ifnar1*^*-/-*^ versus *Ifnar1*^*-/-*^ mice. **(A)** Gene Set Enrichment analysis showing false discovery rate (Q) and normalized enrichment scores of hallmark gene sets most enriched for differentially expressed genes. **(B)** Heat maps reflecting transcript abundance of the top 40 genes in the leading edges, and bottom 20 genes in the trailing edges, of the hallmark “inflammatory response” and “complement” gene sets, and all genes in the hallmark “angiogenesis” gene set. The number of genes in each gene set is shown at the top. **(C)** Venn diagram showing overlap between genes in the leading edges of the hallmark gene sets shown in panel B. **(D)** Volcano plot showing differential expression of genes in HAV-infected *Irf3*^*S1/S1*^*Ifnar1*^*-/-*^ and *Ifnar1*^*-/-*^ mice relative to the liver tissue pathology score as a continuous variable (see S1H in S1 Table). Data were conditioned for sex prior to analysis.

GSEA revealed a strong concordance between the IRF3-regulated genes that were significantly downregulated in infected *Irf3*^*S1/S1*^*Ifnar1*^-/-^ mice versus *Ifnar1*^-/-^ mice and established hallmark gene sets related to inflammatory response, angiogenesis, and complement [25] (**Fig 5A and 5B**). However, there was surprisingly little overlap in the leading edge genes in these three gene sets, suggesting that the enrichment scores for each are driven primarily by different genes **(Fig 5C)**. Normalized enrichment scores were lower, yet still significant with hallmark gene sets for TNF-α signaling via NF-κB and apoptosis (**Figs 5A** and **S4A**). These two hallmark gene sets show greater overlap with the inflammatory response gene set, with *Il1b* (IL-1β) notably present among the top 40 leading edge genes down-regulated in *Irf3*^*S1/S1*^*Ifnar1*^-/-^ mice in each gene set (**S4B Fig**). Thus, despite the substantial overlap in genes induced by HAV in *Irf3*^*S1/S1*^*Ifnar1*^-/-^ and *Ifnar1*^-/-^ mice (**Fig 4D and 4F**), differences in the innate immune responses elicited by infection in these two types of mice involve distinct sets of genes linked to each of the well-defined biological states represented by these specific hallmark gene sets.

The substantial overlap evident in genes induced by HAV in *Irf3*^*S1/S1*^*Ifnar1*^-/-^ and *Ifnar1*^-/-^ mice (**Fig 4D and 4F**) contrasts also with the absence of hepatocellular apoptosis and inflammation in the former (**Fig 3G and 3H**). While this could reflect the globally lower fold-induction of these genes in *Irf3*^*S1/S1*^*Ifnar1*^-/-^ mice, the GSEA suggests it results from differences in the induction of specific genes. Thus, we sought to identify genes with a strong positive correlation between expression level and liver histopathology score in the combined cohort of HAV-infected and naïve *Irf3*^*S1/S1*^*Ifnar1*^-/-^ and *Ifnar1*^-/-^ mice. To accomplish this, liver tissue from each of the 8 infected *Irf3*^*S1/S1*^*Ifnar1*^-/-^ and *Ifnar1*^-/-^ mice was scored for histopathologic changes on a 0–4 scale by a veterinary hepatic pathologist based on the intensity of inflammatory infiltrates and numbers of apoptotic hepatocytes. We then analyzed the data from the these mice, first conditioning for sex, and then identifying genes that were differentially expressed using the pathology score as a continuous variable. This revealed 29 genes for which there was >1.0 log_2_-fold increase in expression, and 63 genes with >0.8 log_2_-fold increase in expression for each 1 unit increase in the recorded pathology score with Q <0.05 **(Fig 5D** and **S1H Table)**. These included transcripts encoding both type 1 and type III IFNs (*Ifnb*, *Ifna4*, *Ifnl3*, *Ifnl2*) and multiple interferon-responsive genes, chemokines, and cytokines (see **Discussion**).

### HAV challenge of *Ifnlr1*^*-/-*^*Ifnar1*^*-/-*^double receptor knockout mice

**Fig 6:**
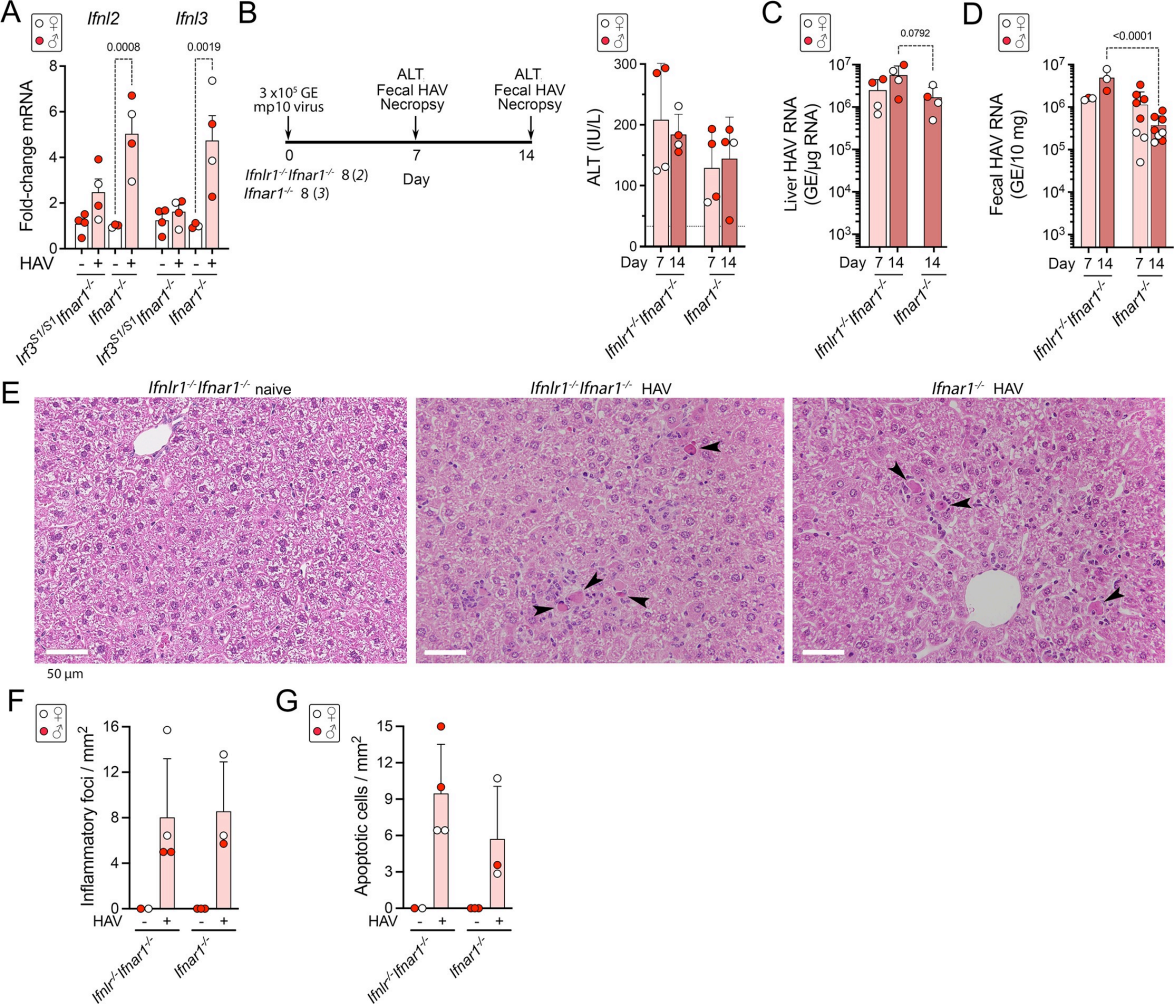
HAV infection in *Ifnlr1*^*-/-*^*Ifnar1*^*-/-*^ double-knockout mice lacking receptors to both type I and III interferon. **(A)** Infection-induced changes in *Ifnl2* and *Ifnl3* transcript abundance in *Irf3*^*S1/S1*^*Ifnar1*^*-/-*^ and *Ifnar1*^*-/-*^ mice determined by RT-qPCR in the experiment outlined in Fig 3A. In all panels, male animals are indicated by solid red symbols, and female animals by open symbols. **(B)** (*left*) Experimental scheme and (*right*) mean serum alanine transaminase (ALT) activities in sera of *Ifnlr1*^*-/-*^*Ifnar1*^*-/-*^ and *Ifnar1*^*-/-*^ mice 7 and 14 days after i.v. inoculation (dpi) of the 3 x10^5^ GE (genome equivalents) HM175-mp10 virus. In each group, 4 infected animals were necropsied on day 7, and 4 on day 14 dpi. **(C)** Mean HAV RNA abundance in liver tissue and **(D)** feces determined by RT-qPCR. Fecal samples from additional *Ifnar1*^*-/-*^ mice infected with the same inoculum were included to demonstrate the range of fecal shedding in these animals. GE = genome equivalents. **(E)** Representative H&E stained sections of liver from HAV-naïve and infected mice 14 dpi. Arrowheads indicate apoptotic hepatocytes, many surrounded by inflammatory immune cells. (**F)** Number of inflammatory foci and **(G)** apoptotic hepatocytes per mm^2^ in H&E-stained liver sections at 14 dpi. Error bars in all panels are s.d.

It was surprising to find a strong correlation between *Ifnl3* and *Ifnl2* expression levels and histopathology **(Fig 5D),** as murine hepatocytes are nonresponsive to type III IFN [26,27]. Nonetheless, RT-qPCR confirmed that these transcripts were minimally induced in infected *Irf3*^*S1/S1*^*Ifnar1*^-/-^ mice compared with *Ifnar1*^-/-^ mice (**Fig 6A**). Thus, to determine whether type III IFN expression contributes directly to HAV-induced liver injury, we challenged mice with dual deficiency of both type I and type III IFN receptors. Groups of 4 *Ifnlr1*^*-/-*^*Ifnar1*^*-/-*^ and *Ifnar1*^*-/-*^ mice were inoculated intravenously with 3 x10^5^ GE mp10 virus. Serum ALT elevations were comparable in the two types of mice at days 7 and 14 p.i. (**Fig 6B**), but HAV RNA levels in both liver and feces were significantly greater in the *Ifnlr1*^*-/-*^*Ifnar1*^*-/-*^ mice than *Ifnar1*^-/-^ mice on day 14 post-infection (**Fig 6C and 6D**). Histopathologic examination revealed similar numbers of apoptotic hepatocytes and similar inflammatory infiltrates in the infected *Ifnlr1*^*-/-*^*Ifnar1*^*-/-*^ and *Ifnar1*^*-/-*^ mice (**Fig 6E and 6G**). Thus, while the additional absence of IFNλ signaling renders *Ifnar1*^*-/-*^ mice somewhat more permissive for HAV infection, it does not reduce liver injury.

## Discussion

Although liver injury in acute hepatitis A is considered generally to result from immune responses to the infection, it remains incompletely understood. The cytotoxic activity of virus-specific CD8+ T cells has been suggested to mediate HAV-induced liver disease [28,29], but more recent studies cast some doubt on this. Virus-specific CD4+ helper cells dominate the adaptive cellular response to HAV in experimentally-infected chimpanzees, whereas CD8+ T cell responses are generally weaker and correlate poorly with both liver injury and virus control [30]. Interleukin 15 (IL-15)-induced bystander activation of CD8+ memory T cells targeting viruses other than HAV has been shown to result in innate-like cytotoxic activity in HAV-infected humans [31]. The cytotoxic activity of bystander-activated T cells correlated better with serum ALT elevations than the cytotoxic activity of virus-specific CD8+ T cells in hospitalized patients with severe disease [31], but it is not known whether this mechanism of liver injury is active in individuals with less severe and more typical infection. Mucosal-associated invariant T (MAIT) cells have also been shown to be activated, and to exert innate-like granzyme B-dependent cytotoxicity in patients with severe disease [32]. In contrast to these proposed cell-mediated mechanisms of liver injury, hepatitis in infected *Ifnar1*^*-/-*^ mice results from cell-intrinsic innate immune responses to the virus [3]. It is possible that no one mechanism accounts for the liver injury in infected humans, and that each of these mechanisms may have a role in producing the spectrum of disease observed in HAV infection.

HAV induces neither hepatocyte apoptosis nor hepatic inflammation in *Mavs*^*-/-*^ mice, despite the quantity of virus in the liver being equal to or greater than that in infected *Ifnar1*^*-/-*^ mice [3] **(Fig 1C and 1D)**. The absence of liver injury in *Mavs*^*-/-*^ mice shows that HAV replication is noncytopathic, and that hepatitis in *Ifnar1*^*-/-*^ mice results from the host response to the infection. *Ifnar1*^*-/-*^ mice recapitulate many of the cardinal features of hepatitis A in humans, including hepatocyte apoptosis and elevated serum ALT levels [3,7]. Apoptotic hepatocytes are surrounded by inflammatory cells, and CD4+ and CD8+ T cells, NK cells, and F4/80+CD11+ macrophages are all increased in number in the liver [3,33]. However, antibody depletion of CD4+ or CD8+ T cells or NK/NKT cells, or clodronate-depletion of phagocytic cells prior to virus challenge does not prevent hepatocellular apoptosis or liver inflammation [3]. Moreover, depleting T cells in mice with established infection leads to increased hepatocyte apoptosis, greater serum ALT elevation, and enhanced inflammation [34]. Like *Mavs*^*-/-*^ mice, *Irf3*^*-/-*^*Irf7*^*-/-*^ mice also show no evidence of liver injury when infected with HAV [3]. Pathogenesis is thus dependent upon signaling through the MAVS-IRF3 axis, likely following sensing of the virus by RLRs including RIG-1 [35].

Recent studies have revealed a key role for IRF3 activation in the pathogenesis of chemically-induced liver disease [10,15]. Following exposure of mice to ethanol or CCl_4_, IRF3 is phosphorylated by TANK-binding kinase 1 (TBK1) secondary to signaling from cyclic GMP-AMP synthase (cGAS) and stimulator of interferon genes protein (STING) [10,14]. *Irf3*^*-/-*^ mice are protected against liver injury following binge exposure to ethanol, whereas *Irf3*^*S1/S1*^ mice with transcriptionally-incompetent IRF3 develop steatosis, ALT elevation and inflammatory cytokine expression [16]. The IRF3 S1 mutant is ubiquitylated and interacts with mitochondrial Bax, inducing apoptosis and liver injury independently of IRF3-mediated transcription [9,16]. In contrast, we found here that HAV-induced inflammation was markedly attenuated in *Irf3*^*S1/S1*^*Ifnar1*^*-/-*^ mice compared to *Ifnar1*^*-/-*^ mice expressing wild-type IRF3 (**Fig 3E–3H**). We cannot rule out a contribution from non-transcriptional IRF3 activity, but our results show that IRF3-mediated transcription is required for liver injury in HAV-infected mice, providing a clear distinction from IRF3-mediated alcohol liver injury.

The contrast between hepatic inflammation in infected *Ifnar1*^*-/-*^ mice and its near complete absence in *Irf3*^*S1/S1*^*Ifnar1*^*-/-*^ mice could reflect quantitatively greater induction of qualitatively similar innate immune response programs including multiple pro-apoptotic and pro-inflammatory mediators (**Figs 4C** and **S4C**). Alternatively, the liver injury in *Ifnar1*^*-/-*^ mice could result from qualitative differences in the host response, with induction of specific genes uniquely dependent upon IRF3 phosphorylation and minimally induced by IRF1 and other transcription factors activated in *Irf3*^*S1/S1*^*Ifnar1*^*-/-*^ mice (**Fig 4E and 4F**). We identified 63 genes for which the level of expression correlated well with liver pathology read blindly by an experienced veterinary hepatic pathologist (**S1H Table**). Many of these genes were minimally or not induced at all in infected *Irf3*^*S1/S1*^*Ifnar1*^*-/-*^ mice. These included genes encoding IFNλ3 and IFNλ2 (**S5B Fig**). However, like type I IFN signaling, type III IFN signaling is not required for hepatocellular apoptosis or liver inflammation, as both occur in infected mice lacking IFNλ receptors (**Fig 6**).

No one gene is likely to be responsible for the liver injury associated with HAV infection in *Ifnar1*^*-/-*^ mice, and many of the genes we found expressed in a manner correlating with liver pathology have been associated previously with apoptotic signaling. These include in particular *Ifi27l2a* and *Ifi27l2b*, which encode the BH3-like IFNα-inducible proteins 27-like 2A/B, also known as ISG12 (**Fig 5D and S1H Table**) [36,37]. There are three ISG12 family members expressed in mice, ISG12A (*Ifi27*), ISG12 (*Ifi27l2a*), and ISG12b2 (*Ifi27l2b*), each of which is significantly induced by HAV infection in *Ifnar1*^*-/-*^ mice (**S5A and S5B Fig**). The physiological functions of these proteins are poorly understood, but both murine and human ISG12 family members are associated with mitochondria and have been implicated in regulating IFN-induced apoptosis [36,38,39]. Human ISG12A (which shares 75% identity with mouse ISG12A) has been linked to the induction of apoptosis in hepatitis C virus (HCV) infected human hepatocytes [37]. Other genes associated with apoptosis include *Lta* (lymphotoxin-alpha), *Tnfrsf9* (TNF receptor superfamily member 9, or CD137), and *Oas1g*, *Oas1a*, and other members of the 2’-5’ oligoadenylate synthetase family.

Finally, it should be noted that previous HAV challenge experiments using *Irf3*^*-/-*^*Irf7*^*-/-*^ mice [3] were potentially confounded by an unintended deletion of a pro-apoptotic gene, *Bcl2l12*, closely juxtaposed to *Irf3* [40]. The *Bcl2l12* gene is intact and normally expressed in *Irf3*^*S1/S1*^*Ifnar1*^*-/-*^ mice (**S5C Fig**). Thus, the absence of liver injury in infected *Irf3*^*S1/S1*^*Ifnar1*^*-/-*^ mice (**Fig 4E**) confirms that the lack of liver inflammation in earlier studies of infected *Irf3*^*-/-*^*Irf7*^*-/-*^ mice [3] resulted from the deletion of *Irf3*, not *Bcl2l12*.

In summary, the studies described here show that IRF3-dependent gene expression promotes hepatocyte apoptosis and liver inflammation during acute HAV infection in mice, and identify sets of IRF3-responsive genes that act together to promote pathogenesis. Similar pathogenetic processes likely drive inflammation of the liver in many humans with acute hepatitis A, and may contribute to both acute and chronic liver injury associated with other hepatotropic viruses.

## Materials and methods

### Ethics statement

The care and use of mice in the experiments described in this report were reviewed and approved by the Animal Care and Use Committee of The University of North Carolina at Chapel Hill, Chapel Hill, North Carolina, or by the Animal Care and Use Committee of the National Institute of Infectious Diseases, Tokyo, Japan.

### Hepatitis A Virus (HAV)

Infections were carried out with mouse-passaged HM175 virus [3]. Inocula were prepared from lysates of liver from infected *Mavs*^*-/-*^ mice after a total of 6 (HM175-mp6) or 10 (HM175-mp10) mouse-to-mouse passages as described previously [3].

### Mice

Mice were bred and housed at the University of North Carolina at Chapel Hill in accordance with the polices and guidelines of the UNC Institutional Animal Care and Use Committee, or at the National Institute of Infectious Diseases in accordance with the policies and guidelines of the NIID Animal Care and Use Committee. C57BL/6 mice were purchased from the Jackson Laboratory. *Ifnar1*^-/-^ mice were provided by J. Sprent of the Scripps Research Institute, or by S. Morikawa of the National Institute of Infectious Diseases, Japan. *Mavs*^-/-^ mice were provided by M. Gale Jr. of the University of Washington, USA. *Ifit*^*L-/-*^ mice with deletion of the complete *Ifit* gene locus [21] and *Irf3*^S1/S1^ mice with transcriptionally-incompetent IRF3 [9] have been described previously. *Ifnar1*^-/-^*Ifnlr*^*-/-*^ mice [27] were provided by P. Staeheli, Institute for Virology, Universitätsklinikum Freiburg. All were bred on a C57BL/6 background for at least 10 generations. *Ifit1*^*L-/-*^*Ifnar1*^-/-^ and *Irf3*^S1/S1^*Ifnar1*^-/-^ mice were crosses between *Ifnar1*^-/-^ mice and *Ifit*^*L-/-*^ mice [21] and *Irf3*^S1/S1^ mice [9], respectively. All mouse genotypes were confirmed by PCR-based genotyping.

### HAV infectious challenge

Mice were intravenously inoculated with the indicated virus inocula at 6–8 weeks of age. Feces were collected from mice housed in individual cages, and serum samples were collected periodically. Tissues were harvested at necropsy and snap frozen on dry ice, stored in RNAlater (Thermo Fisher Scientific) and kept at -80°C, or fixed in 10% neutral phosphate-buffered formalin for 48 hours and stored subsequently in 70% ethanol until processed for histology. All two-way comparisons of infection outcomes in different mouse genotypes were based on use of the same inoculum, identical infection schedules, and data collected from mice infected contemporaneously in the same experiment.

### Alanine Aminotransferase assay

Serum alanine aminotransferase (ALT) activity was measured using the Alanine Aminotransferase Activity Assay Kit (Reitman-Frankel Method, Elabscience) or Alanine Transaminase Colorimetric Activity Assay Kit (Cayman Chemical, Ann Arbor, MI), according to the manufacturers’ recommended procedures.

### Histopathology and immunohistochemistry

Formalin-fixed paraffin-embedded (FFPE) livers were sectioned at 4 μm thickness for histopathology and immunohistochemistry. Sections were stained with hematoxylin and eosin (H&E) and scanned using a Leica Biosystems ScanScope AT2 brightfield line scanner within Translational Pathology Laboratory at University of North Carolina at Chapel Hill. Cleaved caspase 3 staining was accomplished using Dako Autostainer (Dako, Carpinteria, CA). Tissue sections were de-paraffinized and rehydrated prior to antigen retrieval in Pascal presser cooker (Dako) for 30 sec at 123°C in Tris-EDTA buffer. Slides were cooled, rinsed and then treated sequentially with endogenous peroxidase blocker and universal block prior to incubation for 2 hr at room temperature with antibody to cleaved caspase 3 (Cell Signaling, 9661S) diluted 1:100, and 30 min at room temperature with secondary antibody followed by development with 3,3′-diaminobenzidine and counterstain with hematoxylin. Slides were then dehydrated and mounted. Antibodies used in this study are listed in Supporting Information (**S2 Table)**.

### Histopathology scoring

Liver sections were reviewed and scored for pathological changes by a board-certified veterinary pathologist who was blinded to infection status and mouse genotype. Foci of inflammation or hepatocellular apoptosis were enumerated microscopically in 10–20 successive random fields of view using a 40X objective and a 10X ocular (FN22), or in similarly sized fields from high-resolution scanned digital images. Numbers of apoptotic cells, identified by acidophilic cytoplasmic staining and disrupted nuclear morphology in H&E-stained sections or by immunohistochemical staining for cleaved caspase 3, and foci of inflammatory cells were normalized to the area scanned and are reported as foci, or cells, per mm^2^. To assess the differential expression of genes based on pathology as a continuous variable, liver tissues were assigned a pathology score on a 0–4 scale by a veterinary hepatic pathologist based on the intensity of inflammatory infiltrates and numbers of apoptotic hepatocytes.

### *In situ* Hybridization (ISH)

Fluorescent in situ hybridization of formalin-fixed paraffin imbedded tissue sections was carried out using the QuantiGene ViewRNA FFPE Assay (Affymetrix, Santa Clara, CA) as described previously [3].

### High-throughput RNA sequencing and data analysis

RNA was extracted from mouse liver using the *mir*Vana miRNA Isolation Kit (Thermo Fisher, AM1560). Libraries were prepared using the TruSeq Stranded Total RNA Library Prep Gold Kit (Illumina) and sequenced on an Illumina HiSeq 4000 sequencer with pair-end 50 bp setting within the High-Throughput Sequencing Facility of the University of North Carolina at Chapel Hill. Sequencing reads were aligned to the mouse genome mm10 with STAR 2.6 and transcripts quantified using Salmon 0.7 and Gencode vM17. Differential expression analysis was carried out using default settings in the DESeq2 package in R3.6. Transcript counts were normalized using the DESeq2 internal median of ratios method. Genes with base mean read counts <10 were excluded from analysis. Principal component analysis plots were generated by the DESeq2 plotPCA routine after variance-stabilizing transformation of the read counts. Conditioning of sex and batch effect were carried out in DESeq2 by introducing sex and batch as independent co-variants in the design of the experiment as: “design = ~ sex+ batch + condition”. Significance was adjusted for multiple comparisons using the Benjamini-Hochberg method. Gene set enrichment analysis was carried out using normalized read counts for all genes and the GSEA software package: https://www.gsea-msigdb.org/gsea/index.jsp [41,42].

### Quantitative RT-qPCR

Total RNA was extracted from liver using Trizol (Invitrogen), and from feces using the QIAamp Viral RNA Mini Kit (Qiagen). First-strand cDNA was synthesized with Superscript III reverse transcriptase (Invitrogen), and quantitative polymerase chain reaction carried out using Universal SYBR Supermix (Bio-Rad) as described [3]. HAV RNA was quantified against a synthetic RNA standard. Primer sequences are listed in Key Resources.

### Immunoblots

Frozen mouse liver samples were homogenized in the presence of lysis buffer (15% w/v, Invitrogen) containing protease inhibitors (Sigma-Aldrich), 1mM Na_3_VO_4_, and 50mM NaF, clarified by centrifugation twice at 12,000 g for 30 min at 4°C, and stored at -80°C. The samples were subjected to SDS-PAGE and immunoblotting using standard methods. Blots were blocked with Odyssey blocking buffer (LI-COR Bioscience) and probed with the following primary antibodies: IRF1 (8478, 1:500, Cell Signaling), IRF3 (4302, 1:400, Cell Signaling), phospho-IRF3 (Ser396) (4947, 1:200, Cell Signaling), IRF5 (20261S, 1:200, Cell Signaling), β-actin (3700, 1:5000, Cell Signaling). IRDye 680 and 800 secondary antibodies (LI-COR Bioscience) were used to visualize protein bands with an Odyssey two-color detection imaging system (LI-COR, Bioscience).

### Statistical analysis

Statistical tests were carried out using GraphPad Prism 9.0.1 for Mac OS X software. Unless otherwise noted, comparisons between groups used the non-parametric Mann-Whitney test, and one-way or two-way ANOVA. Details of specific statistical tests and experimental design are given in the relevant figure captions. Statistical significance was defined as p<0.05 with appropriate adjustment for multiple tests.

## Supporting information

S1 Fig High-throughput sequencing of RNA from liver tissue of naïve and HAV-infected *Mavs-/-* and *Ifnar1-/-* mice. **(A)** Principal component analysis of RNAseq data from 24 mice in groups of 6 infected and 6 uninfected of each genetic knock-out. First and second components (*left*) represent variance due to differences in sex (42%) and sequencing run (batch, 18%); RNAseq reads from noninfected and infected *Ifnar1*^*-/-*^ mice cluster separately from naïve *Mavs*^*-/-*^ mice based on the 3^rd^ and 4^th^ components (combined contribution to variance = 15%). **(B,C)** Heat maps reflecting (B) differential expression of the 617 genes which were significantly changed (padj<0.05) following infection in either *Mavs*^*-/-*^ or *Ifnar1*^*-/-*^ mice, and (C) differentially expressed genes in liver tissue from (*left*) *Mavs*^*-/-*^ (n = 40 genes) and (*right) Ifnar1*^*-/-*^ mice (n = 579 genes). Scales on the right represent z-score. **(D)** Expanded volcano plot showing fold-change (fc) and significance (padj = adjusted p-value) of transcripts with significant change in abundance in liver tissue from *Mavs*^*-/-*^ mice (see Fig 1G in the main manuscript). **(E)** Average reads mapped in liver tissue from naïve and HAV-infected *Mavs*^*-/-*^ mice for those 25 transcripts with the greatest fold-change in abundance in infected *Ifnar*^*-/-*^ mice (see Fig 1F in the main manuscript). **(F)** Gene set enrichment analysis of transcripts with significant change in abundance in *Ifnar*^*-/-*^ mice. No significant hallmark signatures were identified for tissue from *Mavs*^*-/-*^ mice.
(TIF)

S2 Fig Fluorescent *in situ* hybridization of liver tissue. HAV RNA and *Ccl5* (RANTES) mRNA were probed for in liver tissue from (*top to bottom*) uninfected *Ifnar1*^*-/-*^ mice and HAV-infected *Mavs*^*-/-*^ and *Ifnar*^*-/-*^ mice. Merged fluorescence images are shown on the left, and single-channel images on the right.
(TIF)

S3 Fig High-throughput sequencing of RNA from liver of naïve and HAV-infected *Irf3^S1/S1^Ifnar1^-/-^* and *Ifnar1^-/-^* mice. Liver tissue was harvested 7 days post-inoculation of HM175-mp6 virus. **(A)** Normalized HAV RNA read counts. RPMM = reads per million reads mapped. **(B)** Principal component analysis showing variance related to sex, knockout type, and HAV infection status of animals. **(C)** Correlation plot of fold-change induction of individual genes in *Ifnar1*^*-/-*^ mice infected with HM175-mp10 virus (see Fig 1) versus induction in *Ifnar1*^*-/-*^ mice infected with HM175-mp6 virus (see Fig 4). R^2^ determined by linear regression analysis for all transcripts upregulated >2-fold. **(D)** Volcano plot showing difference (fold-change, fc) and significance of differences in transcript abundance in livers of naive *Irf3*^*S1/S1*^*Ifnar1*^*-/-*^ and *Ifnar1*^*-/-*^ mice. padj = adjusted p-value. **(E)** Venn diagram showing overlap in transcripts downregulated following HAV infection of *Ifnar1*^*-/-*^ and *Irf3*^*S1/S1*^*Ifnar1*^*-/-*^ mice. **(F).** Normalized read counts observed for interferon regulatory factor (IRF) transcripts in livers of naïve *Irf3*^*S1/S1*^*Ifnar1*^*-/-*^ and *Ifnar1*^*-/-*^ mice.
(TIF)

S4 Fig Gene set enrichment analysis of transcripts differentially expressed in HAV-infected *Irf3^S1/S1^Ifnar1^-/-^* versus *Ifnar1^-/-^* mice. **(A)** Heat maps reflecting transcript abundance of top 40 genes in the leading edges, and bottom 20 genes in the trailing edges, of hallmark TNF-α signaling via NF-κB and apoptosis gene sets. The number of genes in each set is shown at the top. **(B)** Venn diagrams showing overlap between the genes in the (*top*) leading edges and (*bottom*) trailing edges of gene sets shown in panel A and the hallmark inflammatory response gene set in Fig 5 in the main manuscript.
(TIF)

S5 Fig *Ifi27* gene family and *Bcl2l12* gene family transcript abundance. Intrahepatic abundance of transcripts encoding ISG12a family members and BCL2L12 in naïve and HAV-infected *Irf3*^*S1/S1*^*Ifnar1*^*-/-*^ and *Ifnar1*^*-/-*^ mice (n = 4 each) 7 days after infection with HM175-mp6 virus. **(A)** Normalized *Ifi27* gene family member read counts from high throughput sequencing. **(B)** Relative transcript abundance determined by RT-qPCR, showing fold-change from uninfected mouse liver. **(C)** Normalized *Bcl2l12* gene family member read counts from high throughput sequencing. Error bars in all panels represent s.d. Significance determined by unpaired t-test.
(TIF)

S1 Table Supporting information tables. (XLSX)

S2 Table Antibodies and other reagents. (PDF)

S3 Table Source data for graphs. (XLSX)
