# Supplementary figures and images for: IRF3-mediated pathogenicity in a murine model of human hepatitis A
Source: PLoS Pathog. 2021 Sep 30;17(9):e1009960. doi: 10.1371/journal.ppat.1009960 (PMC8509855; doi:10.1371/journal.ppat.1009960)

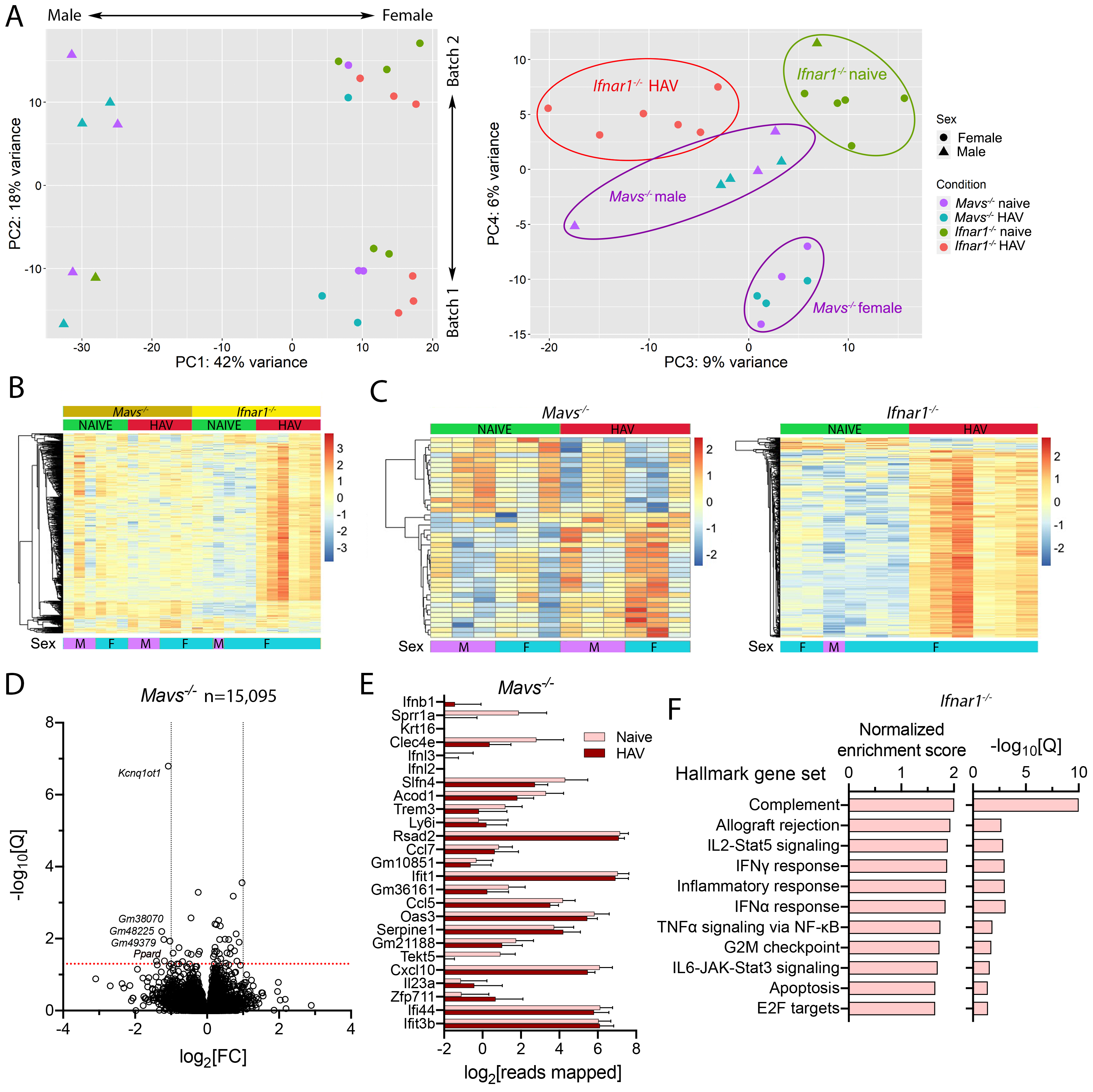

Supplement: S1 Fig — (A) Principal component analysis of RNAseq data from 24 mice in groups of 6 infected and 6 uninfected of each genetic knock-out. First and second components (left) represent variance due to differences in sex (42%) and sequencing run (batch, 18%); RNAseq reads from noninfected and infected Ifnar1-/- mice cluster separately from naïve Mavs-/- mice based on the 3rd and 4th components (combined contribution to variance = 15%). (B,C) Heat maps reflecting (B) differential expression of the 617 genes which were significantly changed (padj<0.05) following infection in either Mavs-/- or Ifnar1-/- mice, and (C) differentially expressed genes in liver tissue from (left) Mavs-/- (n = 40 genes) and (right) Ifnar1-/- mice (n = 579 genes). Scales on the right represent z-score. (D) Expanded volcano plot showing fold-change (fc) and significance (padj = adjusted p-value) of transcripts with significant change in abundance in liver tissue from Mavs-/- mice (see Fig 1G in the main manuscript). (E) Average reads mapped in liver tissue from naïve and HAV-infected Mavs-/- mice for those 25 transcripts with the greatest fold-change in abundance in infected Ifnar-/- mice (see Fig 1F in the main manuscript). (F) Gene set enrichment analysis of transcripts with significant change in abundance in Ifnar-/- mice. No significant hallmark signatures were identified for tissue from Mavs-/- mice. (TIF) [file ppat.1009960.s001.tif]

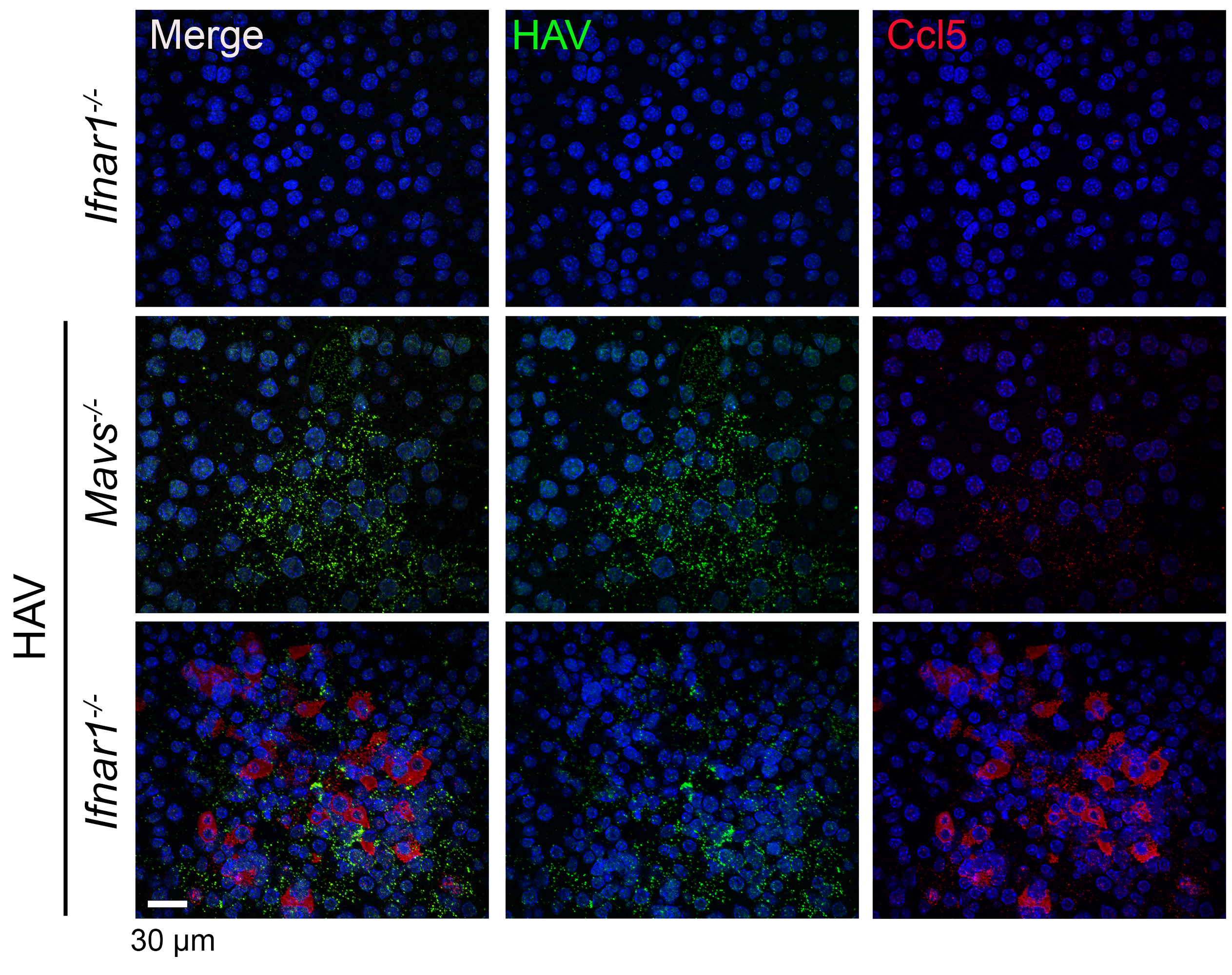

Supplement: S2 Fig — HAV RNA and Ccl5 (RANTES) mRNA were probed for in liver tissue from (top to bottom) uninfected Ifnar1-/- mice and HAV-infected Mavs-/- and Ifnar-/- mice. Merged fluorescence images are shown on the left, and single-channel images on the right. (TIF) [file ppat.1009960.s002.tif]

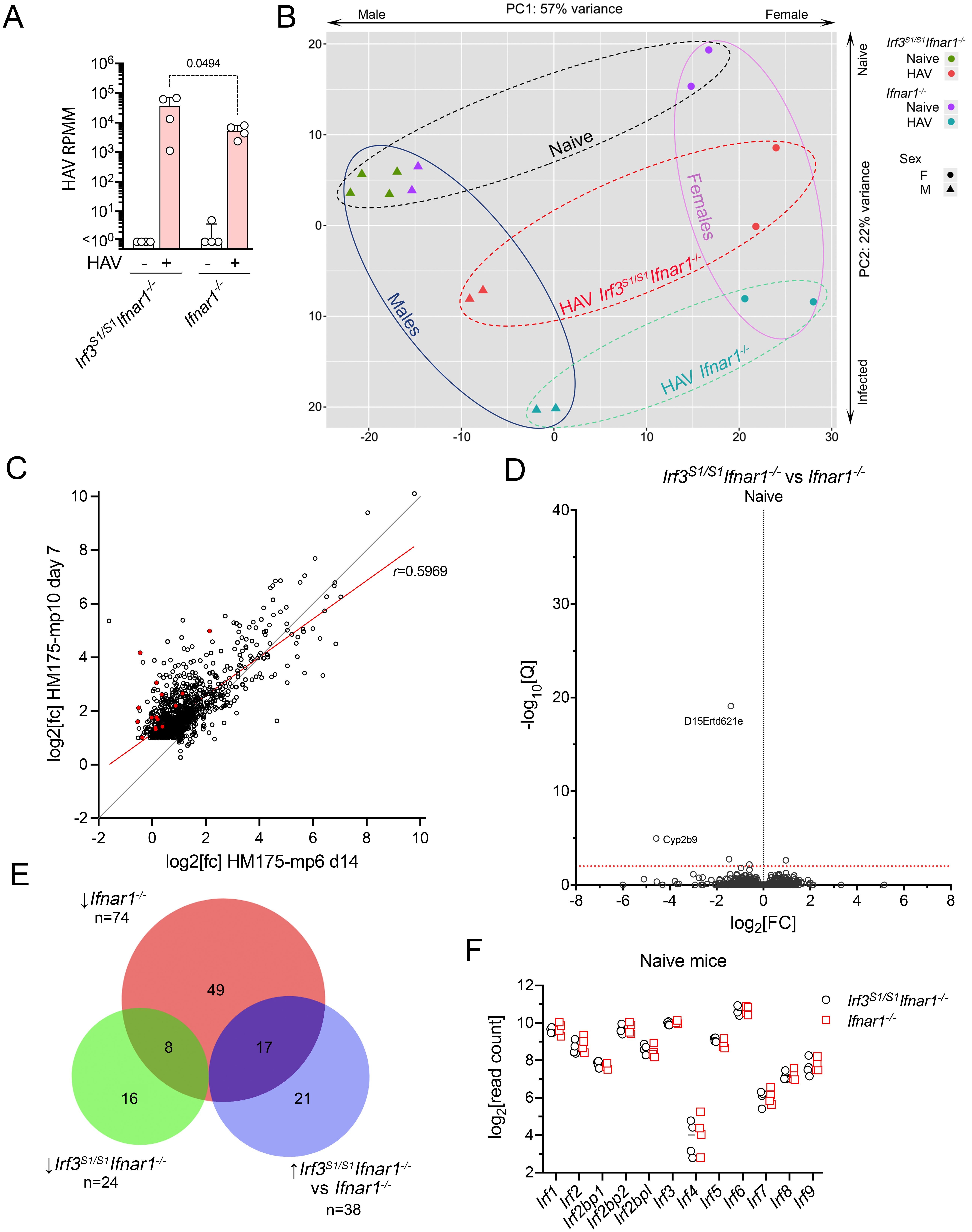

Supplement: S3 Fig — Liver tissue was harvested 7 days post-inoculation of HM175-mp6 virus. (A) Normalized HAV RNA read counts. RPMM = reads per million reads mapped. (B) Principal component analysis showing variance related to sex, knockout type, and HAV infection status of animals. (C) Correlation plot of fold-change induction of individual genes in Ifnar1-/- mice infected with HM175-mp10 virus (see Fig 1) versus induction in Ifnar1-/- mice infected with HM175-mp6 virus (see Fig 4). R2 determined by linear regression analysis for all transcripts upregulated >2-fold. (D) Volcano plot showing difference (fold-change, fc) and significance of differences in transcript abundance in livers of naive Irf3S1/S1Ifnar1-/- and Ifnar1-/- mice. padj = adjusted p-value. (E) Venn diagram showing overlap in transcripts downregulated following HAV infection of Ifnar1-/- and Irf3S1/S1Ifnar1-/- mice. (F). Normalized read counts observed for interferon regulatory factor (IRF) transcripts in livers of naïve Irf3S1/S1Ifnar1-/- and Ifnar1-/- mice. (TIF) [file ppat.1009960.s003.tif]

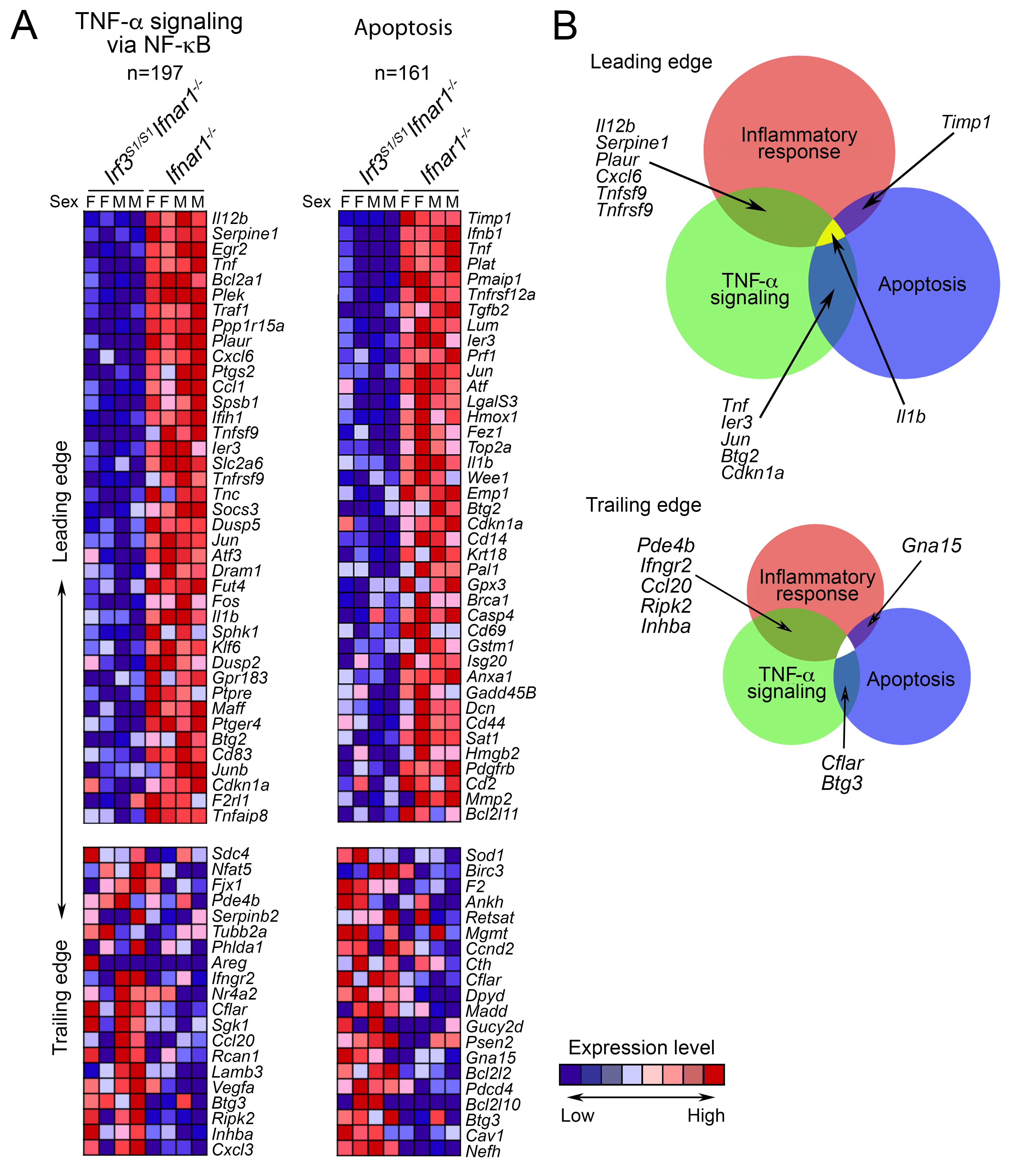

Supplement: S4 Fig — (A) Heat maps reflecting transcript abundance of top 40 genes in the leading edges, and bottom 20 genes in the trailing edges, of hallmark TNF-α signaling via NF-κB and apoptosis gene sets. The number of genes in each set is shown at the top. (B) Venn diagrams showing overlap between the genes in the (top) leading edges and (bottom) trailing edges of gene sets shown in panel A and the hallmark inflammatory response gene set in Fig 5 in the main manuscript. (TIF) [file ppat.1009960.s004.tif]

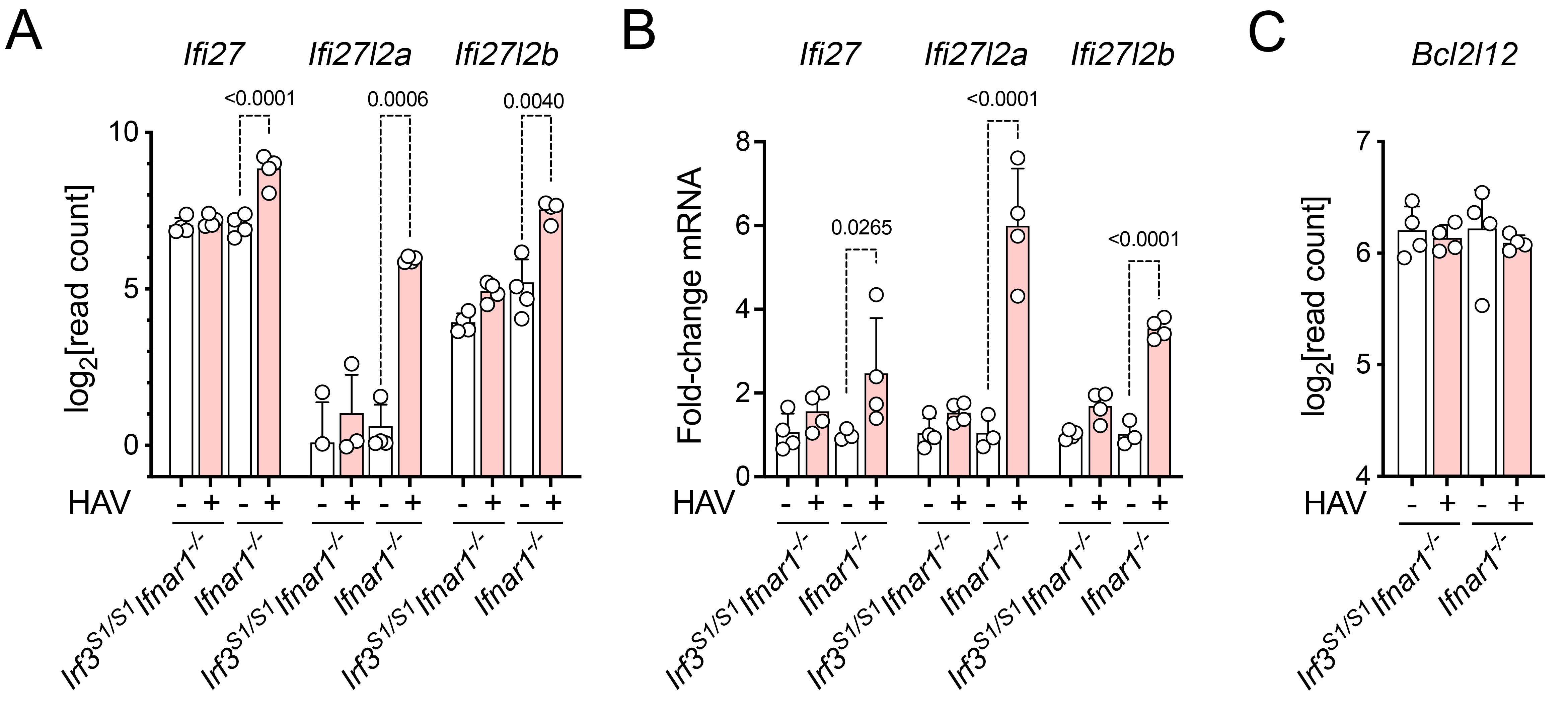

Supplement: S5 Fig — Intrahepatic abundance of transcripts encoding ISG12a family members and BCL2L12 in naïve and HAV-infected Irf3S1/S1Ifnar1-/- and Ifnar1-/- mice (n = 4 each) 7 days after infection with HM175-mp6 virus. (A) Normalized Ifi27 gene family member read counts from high throughput sequencing. (B) Relative transcript abundance determined by RT-qPCR, showing fold-change from uninfected mouse liver. (C) Normalized Bcl2l12 gene family member read counts from high throughput sequencing. Error bars in all panels represent s.d. Significance determined by unpaired t-test. (TIF) [file ppat.1009960.s005.tif]
