# Supplementary material for: IRF3-mediated pathogenicity in a murine model of human hepatitis A
Source: PLoS Pathog. 2021 Sep 30;17(9):e1009960. doi: 10.1371/journal.ppat.1009960 (PMC8509855; doi:10.1371/journal.ppat.1009960)
Supplement: S2 Table — (PDF) [file ppat.1009960.s007.pdf]

S2 Table. Antibodies and Other Reagents

**Antibodies**		
Anti-IRF1	Cell Signaling	Cat #8478
IRF3	Cell Signaling	Cat #4302
Phospho-IRF3	Cell Signaling	Cat #4947
β-Actin	Cell Signaling	Cat #3700
Cleaved Caspase-3	Cell Signaling	Cat #9661S
Phospho-NF-kB p65	Cell Signaling	Cat # 3033
IRF7	Invitrogen	Cat # PA520280
Rabbit IRDye 680 secondary antibody	LI-COR Bioscience	Cat #926-32211
Rabbit IRDye 800 secondary antibody	LI-COR Bioscience	Cat #926-32212
**Chemical Reagents**		
Tissue Extraction Reagent	Invitrogen	Cat #FNN0071
Protease Inbihitor Cocktail	Sigma-Aldrich	Cat #P8340
PhosSTOP	Sigma-Aldrich	Cat #4906845001
cOmplete Protease Inhibitor Cocktail	Roche	Cat #11697498001
SDS-PAGE gel	Bio-Rad	Cat #4568086
Precision Plus Protein Standards	Bio-Rad	Cat #1610347
Odyssey blocking buffer	LI-COR Bioscience	Cat #92770001
RIPA buffer	ThermoFisher	Cat #89900
RnaseZap	Ambion	Cat #AM9780
TRIzol Reagent	Invitrogen	Cat #15596026
**Commercial Assays**		
QIAamp Viral RNA Mini Kit	Qiagen	Cat #52906
RNease Mini Kit	Qiagen	Cat #74160
SuperScript III First-Strand Synthesis SuperMix	Invitrogen	Cat #11752-050
iTaq Universal SYBR Green Supermix	Bio-Rad	Cat #1725121
Serum Alanine Aminotransferase Activity kit	Elabscience	Cat #EBCK235M

**Primers**	**Forward (5'–3')**	**Reverse (5'–3')**
Ccl5	GCTGCTTTGCCTACCTCTCC	TCGAGTGACAAACACGACTGC
Tnfa	CATCTTCTCAAAATTCGAGTGACAA	TGGGAGTAGACAAGGTACAACCC
Isg15	GGGGTAACGATTTCCTG	CATAGATGTTGCTGTGGC
Ifng	GGCAAAAGGATGGTGACATGA	ATCTCTTCCCCACCCCGAAT
Ifnb	GATGACGGAGAAGATGCAGAAG	ACCCAGTGCTGGAGAAATTG
Ifnl2	GTGCAGTTCCCACCTCTTCC	GGCTGAGTCAGTCATGTTCTC
Ifnl3	GTGCAGTTCCCACCTCATCT	GGCTGAGTCATTTATGTTCTC
Ifi27	TTGGAGAGGAACCTGCTTTG	CCTTCCTTTCTCTGCCTTCTT
Ifi27l2a	CGTGACTGAAGACAGCCAATA	CCTTCCATCACTGAGTGTTTCT
Ifi27l2b	CACAGGAGCCTCCAAGTTATG	GAACCAGTGCATCTTGGTACT
Actin	AGTGTGACGTTGACATCCGT	TGCTAGGAGCCAGAGCAGTA
